# Adaptive evolution and phylogeny of cerithioid gastropods with six new mitogenomes

**DOI:** 10.1038/s41598-025-30310-z

**Published:** 2025-12-02

**Authors:** Cho Rong Shin, Eun Hwa Choi, Ui Wook Hwang

**Affiliations:** 1 Department of Biomedical Convergence Science and Technology, Kyungpook National University, Daegu, 41566 South Korea; 2 Department of Biology Education, Teachers College and Institute for Phylogenomics and Evolution, Kyungpook National University, Daegu, 41566 South Korea; 3 Department of Advanced Bioconvergence, Kyungpook National University, Daegu, 41566 South Korea; 4 Institute for Korean Herb-Bio Convergence Promotion, Kyungpook National University, Daegu, 41566 South Korea; 5 Phylomics Inc., Daegu, 41910 South Korea

**Keywords:** Cerithioidea, Mitochondrial genome, Phylogenomics, Adaptive evolution, ND6, Positive selection, Evolutionary biology, Phylogenetics

## Abstract

Cerithioidea (Caenogastropoda: Gastropoda) represents a diverse superfamily of gastropods that inhabit marine, brackish, and freshwater environments worldwide. Despite this broad ecological distribution, their evolutionary history and phylogenetic relationships remain incompletely understood. Here, we report six newly sequenced mitochondrial genomes—two from *Batillaria* (Batillariidae) and four from *Cerithidea, Cerithideopsis*, and *Pirenella* (Potamididae)—to clarify cerithioidean phylogeny and investigate signals of adaptive evolution. Phylogenetic analyses consistently supported the monophyly of Cerithioidea and its subdivision into two major lineages. However, family-level relationships remained partially ambiguous. Batillariidae clustered with Pachychilidae, and Potamididae appeared as sister to Semisulcospiridae in most trees, underscoring the need for broader taxonomic sampling to refine these evolutionary relationships. Divergence-time estimates indicate that major lineages originated during periods of elevated global temperatures, with diversification accelerated by subsequent environmental changes, highlighting the influence of historical climate events on expansion and habitat shifts. Selection analyses reveal ND6 as a hotspot of positive selection, particularly within disordered protein regions that may enhance flexibility under environmental stress. These findings underscore how historical climate events and habitat fluctuations have influenced mitochondrial genome evolution in euryhaline gastropods, offering a refined phylogenetic framework for Cerithioidea and providing insights into the adaptive mechanisms underlying their expansive ecological range.

**Supplementary Information:**

The online version contains supplementary material available at 10.1038/s41598-025-30310-z.

## Introduction

The superfamily Cerithioidea Fleming, 1822 (Caenogastropoda, Gastropoda, Mollusca) is among the most taxonomically and ecologically diverse caenogastropod lineages, being comprised by over 1,500 extant species across 22 families and 175 genera worldwide^1^. These snails inhabit a wide range of aquatic environments, including marine, brackish, and freshwater environments, throughout tropical and temperate regions^2^. Their remarkable ecological breadth and morphological diversity make them a key group for studying evolutionary transitions in gastropods. As one of the basal clades within Caenogastropoda, which includes most modern gastropod species^3^, Cerithioidea provides critical insights into the early diversification of this major molluscan subgroup.

Phylogenetic analyses integrating morphological and molecular datasets have revealed multiple independent transitions between marine and non-marine environments within Cerithioidea^2^. Despite this, familial relationships within this superfamily remain incompletely resolved, and deeper divergences within the group are poorly understood. Morphology-based studies^4,5^ have enhanced our understanding of cerithioidean relationships; however, high phenotypic plasticity has often led to inconsistent or conflicting topologies. Lydeard et al.^6^ produced the first comprehensive molecular phylogeny of Cerithioidea, using partial 16S rRNA sequences to investigate inter-familial relationships. Subsequently, Strong et al.^2^ further analyzed these relationships incorporating 16S rRNA, 28S rRNA, and morphological data, proposing three major cerithioidean clades. However, both studies struggled to resolve the monophyly of certain clades due to limited phylogenetic resolution and low support values. Furthermore, Forestello et al.^7^ reexamined cerithioidean phylogeny by incorporating additional 16S rRNA and 28S rRNA data using maximum likelihood (ML) and Bayesian inference (BI) frameworks. Although their analysis confirmed the monophyly of several previously unresolved groups, some relationships remained ambiguous, and certain nodes showed low support values. Collectively, these findings underscore the need for more comprehensive molecular data, particularly complete mitochondrial genomes (mitogenomes), to clarify unresolved evolutionary relationships within Cerithioidea.

In recent years, mitogenomes have emerged as a powerful tool in invertebrate systematics and evolutionary biology^8,9^. Due to their relatively conserved structure and gene content, mitogenomes provide extensive phylogenetic information and enable the detection of lineage-specific features, such as gene rearrangements and unique substitution patterns^10^. Although recent studies have identified novel gene orders and other evolutionary signatures in cerithioidean mitogenomes^11,12^, taxonomic sampling remains limited across several key lineages. Moreover, growing evidence suggests that positive selection on mitochondrial protein-coding genes (PCGs) can drive adaptations in response to fluctuating environmental conditions, particularly temperature and salinity gradients, among marine and brackish invertebrates^13,14^. Mitochondrial genes, which play a critical role in oxidative phosphorylation, are subject to selective pressures that optimize metabolic efficiency under varying ecological constraints^15^. For instance, several marine invertebrate lineages show signs of positive selection in genes such as ATP6, ATP8, and NADH dehydrogenase subunits, likely reflecting adaptations to maintain respiratory efficiency under challenging or novel conditions^16^. These findings support the notion that mitochondrial adaptations can evolve relatively rapidly, offering a selective advantage in environments characterized by fluctuating energetic demands. Investigating similar patterns in Cerithioidea may reveal how historical and contemporary selective pressures have influenced mitochondrial function in euryhaline gastropods, promoting resilience and diversification across marine and brackish environments.

Here, we present the complete mitochondrial genomes of two batillariid and four potamidid species to help resolve phylogenetic relationships within Cerithioidea and to examine patterns of molecular evolution in mitochondrial PCGs. By integrating molecular phylogenetics with evolutionary rate analyses, we aim to reconstruct the evolutionary history of these lineages and examine how long-term environmental changes may have influenced their diversification. Ultimately, this work enhances our understanding of mitogenome evolution and lineage diversification in euryhaline gastropods.

## Results

### Mitochondrial genomes and organization of six cerithioid species

**Fig. 1:**
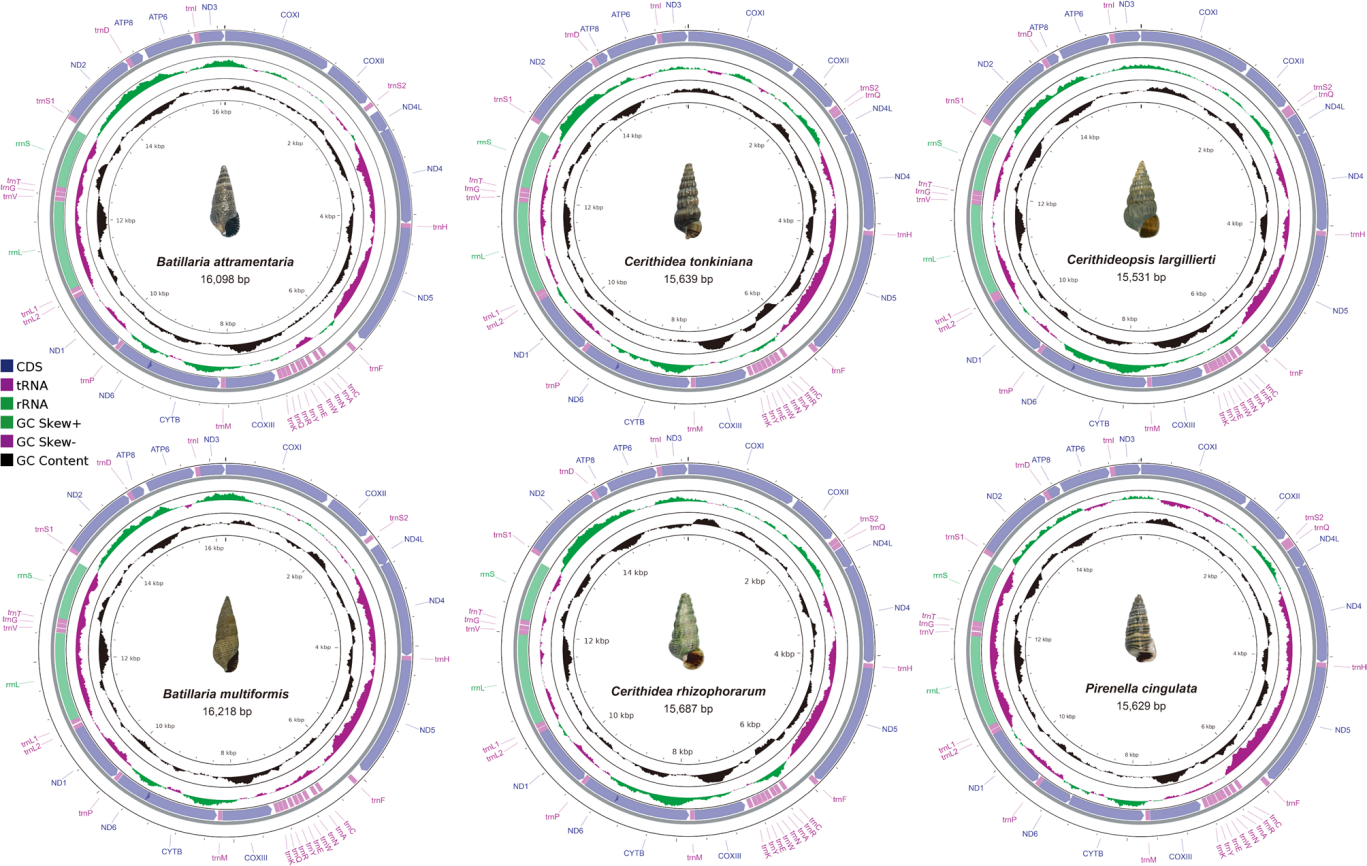
Mitochondrial genome maps of six cerithioidean species. The central image shows a specimen of each species. Circular maps illustrate genomic features: (1) the black ring indicates GC content; (2) the green-purple ring shows GC skew values, where green/purple color intensity reflects the degree of skew; (3) the outer and inner rings depict the arrangement of genes. Genes on the outer ring are encoded on the forward strand, while genes on the inner ring reside on the reverse strand. Different colors denote protein-coding genes (PCGs), ribosomal RNAs (rRNAs), and transfer RNAs (tRNAs).

The complete mitochondrial genomes of the two batillariid species, *Batillaria attramentaria* and *B. multiformis*, measured 16,098 bp and 16,218 bp, respectively (Fig. 1). Both species exhibited the typical gene complement of molluscan mitogenomes, consisting of 13 PCGs, 22 transfer RNA (tRNA) genes, and two ribosomal RNA (rRNA) genes^10,17^. Variation in genome length between the two species was minimal within coding regions; observed differences were primarily limited to non-coding regions, particularly the putative control region. In both *Batillaria* species, all PCGs initiated with ATG and terminated with either TAA or TAG (Supplementary Tables S2–S3). An extended A + T-rich region was identified between *trnF* and *trnC*, consistent with non-coding control regions described in other gastropods. Both species exhibited a 7 bp overlap between *nad4l* and *nad4,* and 47 bp overlap between *cytb* and *nad6* (Supplementary Tables S2–S3). The complete mitochondrial genomes of the four potamidid species measured 15,639 bp in *Cerithidea tonkiniana*, 15,687 bp in *C. rhizophorarum*, 15,531 bp in *Cerithideopsis largillierti*, and 15,632 bp in *Pirenella cingulata* (Fig. 1). All four species utilized ATG as a start codon, except for *nad4* in *P. cingulata* (GTG) and *nad6* in *C. rhizophorarum* (ATT). All PCGs terminated with either TAA or TAG (Supplementary Tables S4–S7), and gene lengths were generally conserved across species, with the exception of *nad6*. Three species contained a 543 bp-long *nad6*, whereas *P. cingulata* possessed a shorter *nd6* (507 bp), consistent with *P. pupiformis*^11^. Similar to Batillariidae, gene overlaps were observed in Potamididae, including 7 bp overlap between *nad4l* and *nad4,* and 38 bp overlap between *cytb* and *nad6*. However, in *P. cingulata*, only a 1 bp-long overlap was observed between *cytb* and *nad6*, whereas the 7 bp overlap between *nad4l* and *nad4* remained. All four potamidid species also contained a long non-coding region between *trnF* and *trnC* (Supplementary Tables S4–S7).

Despite the generally conserved arrangement of PCGs, Batillariidae and Potamididae exhibited differences in the positioning of certain tRNA genes. In Batillariidae, *trnR* is located on the light strand between *trnC* and *trnQ*, whereas in Potamididae it is situated between *trnC* and *trnA*. Additionally, *trnQ* is positioned between *trnR* and *trnK* on the light strand in Batillariidae, but occurs between *trnS2* and *nad4l* on the heavy strand in Potamididae. Most tRNA genes formed the typical cloverleaf structure, including the amino acid acceptor stem, TψC stem, anticodon stem, and DHU stem (Supplementary Figs. S1–S6). The two batillariid species exhibited identical nucleotide lengths for all tRNA genes and shared identical sequences for *trnC*, *trnL2*, *trnR*, *trnS1*, *trnY*, *trnW*, and *trnN* (Supplementary Figs. S1–S2). In contrast, the four potamidid species showed minor length variations (1–3 bp) in several tRNAs, and *trnS1* exhibited a truncated structure lacking a DHU arm (Supplementary Figs. S3–S6).

The overall nucleotide compositions of the complete mitochondrial genomes and the 13 PCGs across the six cerithioidean species are summarized in Table 1. All species demonstrated a pronounced A + T bias (62.51–65.56%), consistent with patterns observed in other molluscan mitogenomes^14,18^. AT skew values were negative in all species, indicating a higher proportion of thymine (T) than adenine (A). GC skew values were predominantly negative, except in *C. rhizophorarum* and *Ce. largillierti*, which exhibited slightly positive values, suggesting a marginal excess of guanine (G) over cytosine (C). Within PCG regions, the A + T content remained high (62.30–66.61%), with even more negative AT skew values, reflecting a stronger T bias in coding sequences, a pattern frequently reported in Caenogastropoda^17,19^.

**Table 1:** Nucleotide composition of the complete mitochondrial genomes and protein-coding genes (PCGs) of six cerithioidean species.

	Total mitochondrial genome					PCGs				
	Length(bp)	A + T (%)	G + C (%)	AT skew	GC skew	Length(bp)	A + T (%)	G + C (%)	AT skew	GC skew
*B. multiformis*	16,098	65.56	34.44	− 0.0764	− 0.0191	11,307	64.31	35.65	− 0.2225	− 0.0265
*B. attramentaria*	16,218	65.21	34.79	− 0.0726	− 0.0351	11,307	64.99	35.01	− 0.2186	− 0.0265
*C. tonkiniana*	15,639	63.16	36.84	− 0.0711	− 0.0036	11,283	62.32	37.68	− 0.2076	− 0.0289
*C. rhizophorarum*	15,687	62.51	37.49	− 0.0773	0.0145	11,283	64.79	35.21	− 0.2194	− 0.0239
*Ce. largillierti*	15,531	64.43	35.57	− 0.0796	0.0072	11,283	66.61	36.39	− 0.1924	− 0.0360
*P. cingulata*	15,629	63.08	36.92	− 0.0481	− 0.0487	11,247	62.30	37.70	− 0.2028	− 0.0307

**Fig. 2:**
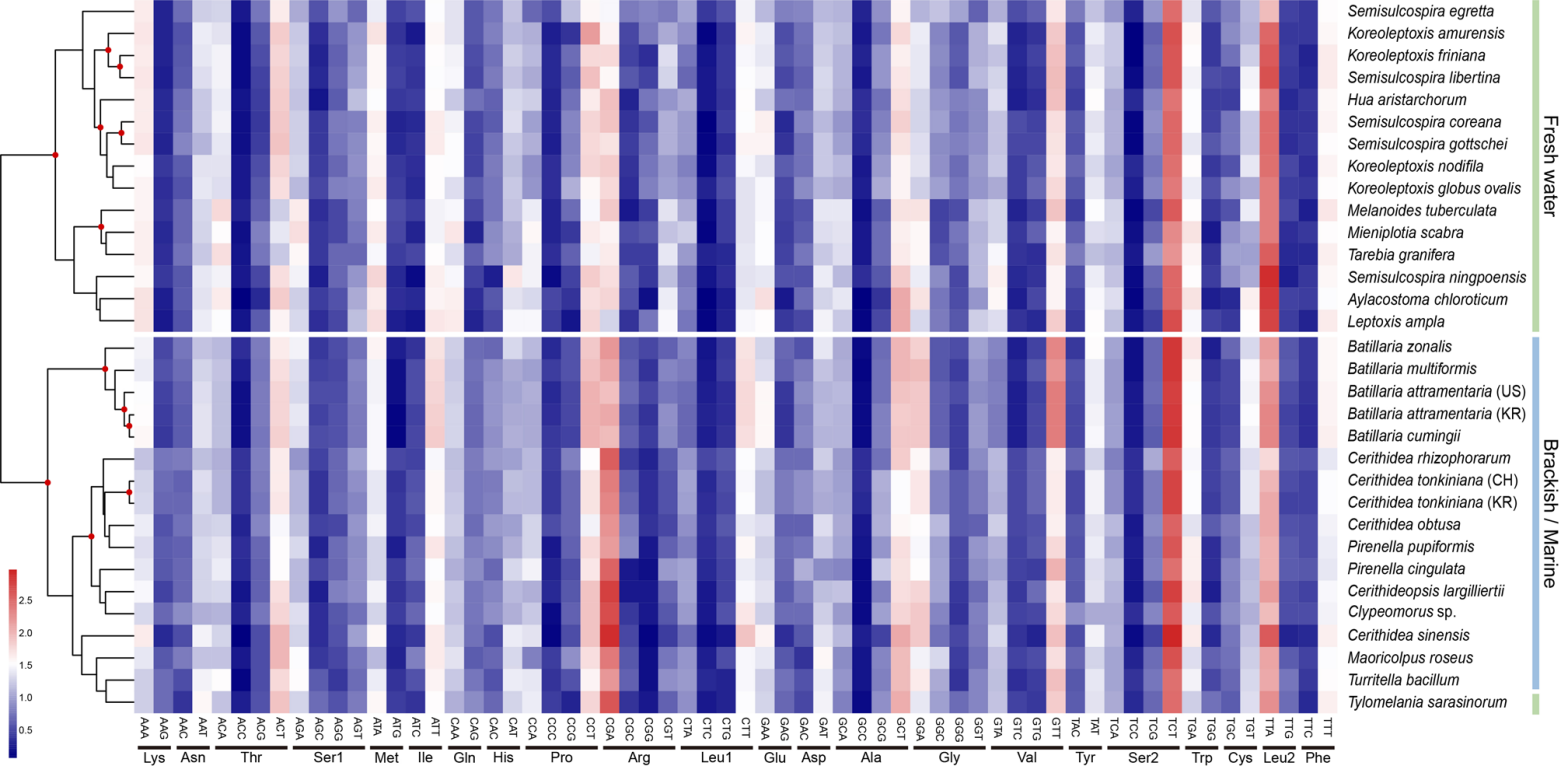
Heatmap of Relative Synonymous Codon Usage (RSCU) for 13 mitochondrial protein-coding genes from Cerithioidea. Data are based on six Cerithioidea mitochondrial genomes. The dendrogram above the heatmap clusters species according to codon usage similarity, using Ward’s method and Euclidean distance. Red dots on branches mark clusters with approximately unbiased (AU) p-values > 95%, indicating strong statistical support. Species labels are color-coded by habitat type (freshwater, brackish/marine) to highlight potential associations between codon usage patterns and environmental conditions.

To assess codon usage across species, a heatmap of relative synonymous codon usage (RSCU) values with a hierarchical clustering dendrogram was generated (Fig. 2). Overall, the six cerithioidean species displayed similar codon usage patterns, with a marked preference for A or T at the third codon position. Despite minor exceptions, the dendrogram generally grouped freshwater and marine species separately, suggesting potential associations between codon usage bias and habitat type, as previously reported^20^.

### Phylogenetic relationships of Cerithioidea

**Fig. 3:**
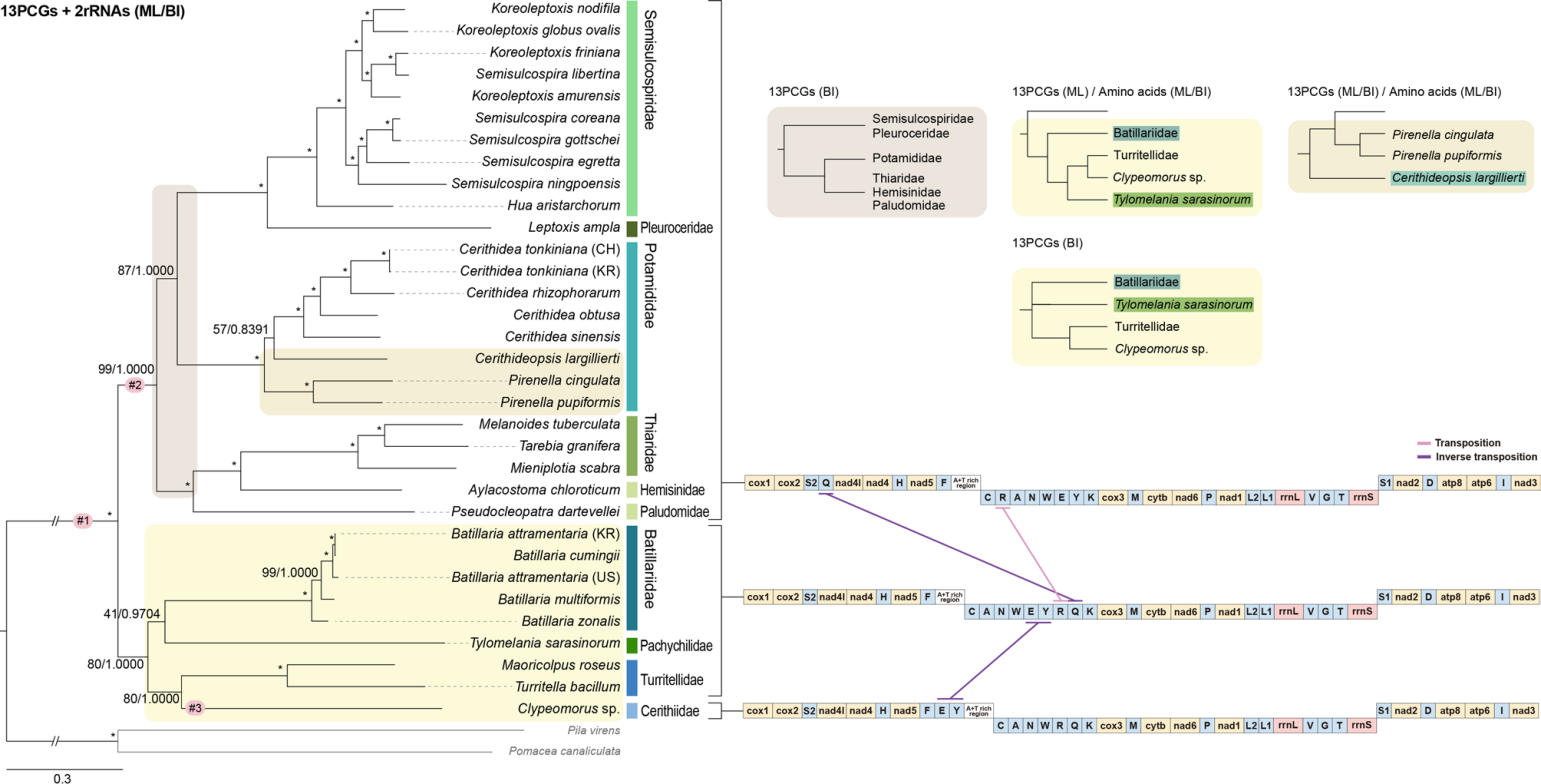
Phylogenetic trees of Cerithioidea inferred from mitochondrial genome sequences. Trees were reconstructed from 13 protein-coding genes (PCGs) and two rRNA genes for 33 cerithioidean species, with two caenogastropod species as outgroups. Maximum Likelihood (ML) and Bayesian Inference (BI) analyses were performed on the nucleotide dataset based on 13 PCGs and 2 rRNAs. ModelFinder 2.2.0 selected the best-fit substitution models, and ML trees were generated via IQ-TREE with 10,000 ultrafast bootstrap replicates. BI trees were inferred using MrBayes (two runs of 10 million generations, sampling every 1,000). Convergence was assessed by the mean standard deviation of split frequencies (< 0.01). The final tree was visualized in FigTree v1.4.4. Nodes labeled #1–#3 indicate branches under positive selection as identified by the aBSREL analysis. Colored boxes highlight alternative topologies observed in different datasets.

Phylogenetic trees reconstructed using ML and BI approaches yielded largely congruent topologies across the three datasets (PCGs + rRNAs, PCGs only, amino acids). The resulting relationships were consistent among analytical methods (Fig. 3). All major cerithioidean families, Batillariidae, Potamididae, and Semisulcospiridae, emerged as strongly supported, monophyletic clades. However, certain taxa adjacent to Batillariidae (e.g., Turritellidae, Cerithiidae, Pachychilidae) exhibited unstable placements and weak support values. *Turritella sarasinorum* formed a sister group to Batillariidae in the PCGs + rRNAs trees; however, it appeared paraphyletic in the ML tree using PCGs and in the amino acid-based analysis and collapsed in BI tree based on PCGs. Specifically, the PCGs + rRNAs trees supported the topology (*Batillariidae* + *Pachychilidae*) + (*Turritellidae* + *Cerithiidae*), whereas the other trees indicated ((*Batillariidae* + ((*Turritellidae* + *Cerithiidae*) + *Pachychilidae*))). In the BI tree based on PCGs, these families formed paraphyletic assemblages with low support. Although certain nodes were weakly supported, Potamididae consistently appeared as the sister group to Semisulcospiridae across nearly all datasets, except in the BI tree derived exclusively from PCGs, where Potamididae instead clustered with ((*Thiaridae* + *Hemisinidae*) + *Paludomidae*), albeit with very low bootstrap support. Within Potamididae, *Cerithidea* and *Pirenella* were both monophyletic, though interspecific relationships were somewhat unstable, particularly involving *Ce. largillierti* and *P. cingulata*. In the PCGs and amino acid-based trees, *Ce. largillierti* occupied the outermost position within Cerithidea, whereas *Pirenella* emerged as the basal taxon in the PCGs + rRNAs dataset.

Although the arrangements of PCG appeared largely conserved throughout Cerithioidea, tRNA organization varied at the earliest divergence points. In Turritellidae, Cerithididae, Pachychilidae, and Batillariidae, *trnQ* was located between *trnR* and *trnK*. In contrast, Semisulcospiridae, Potamididae, Thiaridae, Hemisinidae, and Paludomidae positioned *trnQ* between *trnS2* and *nad4l*. Additionally, the location of *trnR* differed: it was found between *trnY* and *trnQ* in Turritellidae, Pachychilidae, and Batillariidae, and between *trnW* and *trnQ* in Cerithiidae (due to an inverted transposition, *trnE*–*trnY*). In Semisulcospiridae, Potamididae, Thiaridae, Hemisinidae, and Paludomidae, *trnR* was positioned between *trnC* and *trnA* (Fig. 3).

### Adaptive evolution in mitochondrial protein-coding genes

**Fig. 4:**
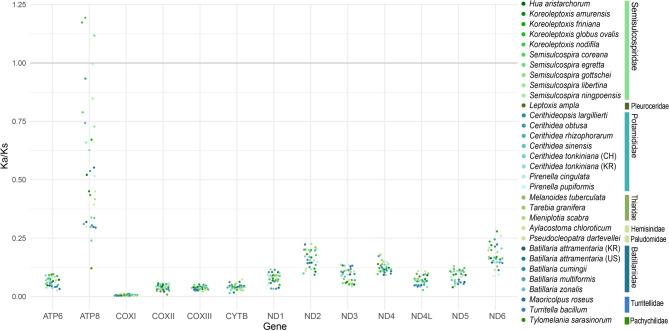
Ka/Ks plot depicting selection pressure across 13 mitochondrial PCGs in Cerithioidea. For each of the 13 mitochondrial protein-coding genes, the ratio of nonsynonymous substitutions (Ka) to synonymous substitutions (Ks) was calculated. Ka/Ks > 1, suggesting potential signals of positive selection, are shown.

Ka/Ks ratios (ω) were used to evaluate selective pressures on the 13 mitochondrial PCGs, with *Clypeomorus* sp. (Cerithiidae) serving as the reference (Fig. 4). A Ka/Ks ratio greater than 1 suggests potential positive selection, whereas a ratio close to 1 indicates neutral evolution, and a ratio less than 1 reflects purifying (negative) selection^21^. Except for *atp8*, which exhibited Ka/Ks ratios greater than 1 in two semisulcospirid species (*Koreoleptoxis nodifila* and *Semisulcospira egretta*) and one pleurocerid species (*Leptoxis ampla*), all other PCGs showed Ka/Ks ratios below 1, implying strong purifying selection. *cox1* exhibited the lowest mean Ka/Ks (approximately 0.007), reflecting a high degree of conservation. Among the NADH dehydrogenase genes, *nad6* showed slightly elevated Ka/Ks values, but remained under purifying selection.

HyPhy analysis revealed pervasive purifying selection across all 13 PCGs in Cerithioidea. FUBAR detected predominantly neutral or negative selection, although two codons in *nad6* suggested possible positive selection. MEME identified six codons under episodic positive selection, including three also highlighted by FUBAR (Table 2). CodeML analysis (PAML) provided further support for weak positive selection, with significant likelihood ratio test results for M0 vs. M3, M1a vs. M3, and M7 vs. M8 (*p* < 0.005), but not for M1a vs. M2a (*p* > 1). Although BEB analysis suggested an overall trend toward positive selection, no individual codon met the BEB significance threshold (*p* > 1).

**Table 2:** Codons under positive selection inferred from site and branch-site models based on mitochondrial protein-coding genes in Cerithioidea.

Gene	Site models		Branch-site models		
	FUBAR	MEME	BEB analysis (M2a)		
	Codon (PP)	Codon (*p* value)	Branch #1 (PP)	Branch #2 (PP)	Branch #3 (PP)
*cox2*					2259 (0.991)
*nad4*					2871 (0.994)
					2904 (0.984)
					3075 (0.984)
		3306 (0.048)			
			3504 (0.964)		
			3528 (0.989)		
					3671 (0.974)
					4029 (0.984)
*nad5*					4192 (0.990)
			4264 (0.988)		
			4303 (0.980)		
		**5647 (0.002)**			**5647 (0.960)**
*cytb*			8146 (0.982)		
				9058 (0.957)	
*nad6*	**9236 (0.918)**	**9236 (0.020)**			
		9239 (0.033)			
	**9242 (0.965)**	**9242 (0.025)**			
	**9260 (0.984)**	**9260 (0.017)**			
					9384 (0.961)
			9402 (0.986)		
					9437 (0.966)
					9446 (0.968)
			9477 (0.997)		
			9480 (0.976)		
*nad1*					10,013 (0.989)
			10,044 (0.963)		
			10,494 (0.992)		
					10,503 (0.986)
*nad2*			13,662 (0.998)		
			13,858 (0.985)		
			14,410 (0.961)		
					14,509 (0.979)
			14,536 (0.990)		
					14,554 (0.966)
*atp8*			14,852 (0.964)		

Codon positions refer to the nucleotide position (start codon numbers) in the *B. attramentaria* mitochondrial genome assembled in this study (PV619093). Branches showing signals of positive selection (*p* < 0.05) were initially identified using the aBSREL method and subsequently designated as foreground branches in the branch-site model of CodeML. Codons with posterior probability values exceeding 1.0 in the BEB analysis under the site model (M8), which are considered unreliable estimates, were excluded from the table.

Significance of bold for positively selected sites was assessed using FUBAR (PP > 0.90), MEME (*p* < 0.05) in Datamonkey, and BEB in CodeML (PP > 0.95).

The aBSREL method identified three branches experiencing episodic diversifying selection (*p* < 0.05), which were subsequently examined using branch-site models in CodeML to detect positively selected codons. Several mitochondrial PCGs contained positively selected sites. In Branch #1, broadly separating freshwater and marine species, most sites under selection were located in *nad2* and *nad6*. Branch #2, which marks the divergence of Semisulcospiridae and Potamididae from other taxa, exhibited one positively selected codon in *cytb*. Branch #3, marking the divergence of Cerithiidae, displayed the highest number of positively selected sites, primarily in *nad2*, *nad4*, and *nad6*. One codon in *nad5* (no. 5647) identified by MEME as episodically selected was also confirmed in Branch #3.

**Fig. 5:**
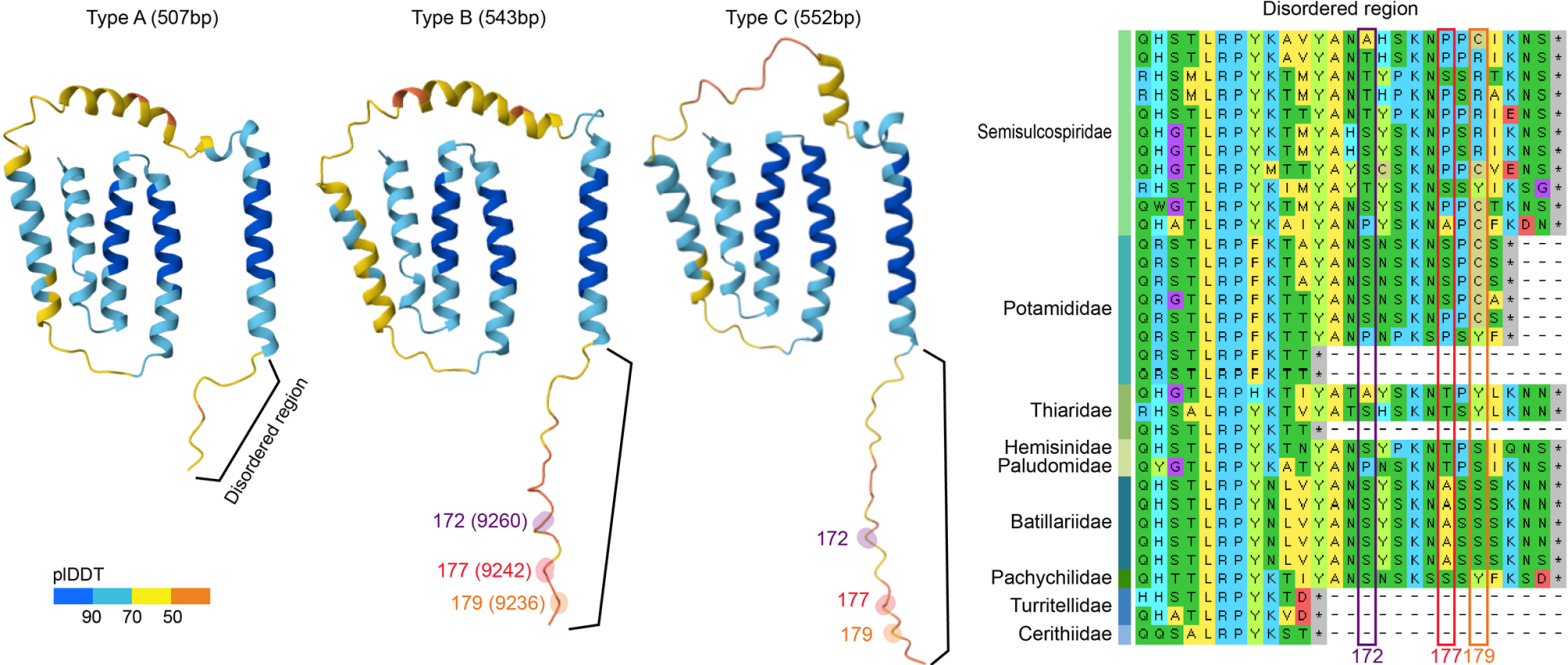
Predicted 3D structure and disordered regions of the ND6 protein from Cerithioidea. The ND6 protein was modeled using AlphaFold, with structural confidence indicated by a color gradient (blue = higher confidence, yellow to red = lower confidence or disordered regions). The C-terminal portion exhibits an extended, disordered region implicated in regulatory flexibility. Sites under positive selection are highlighted on the 3D model and boxed in the sequence.

Although certain codons were identified by only a single method (MEME, FUBAR, or BEB), sites were considered functionally significant only if supported by at least two independent approaches^22^. Three codons in *nad6* consistently appeared in both FUBAR and MEME analyses, prompting further structural analysis (Fig. 5). AlphaFold predictions revealed that the protein’s fundamental α-helical and transmembrane domains were conserved, whereas disordered regions exhibited some variation. These findings suggest that although the fundamental structure of *nad6* remains stable, adaptive changes may occur within flexible regions, reflecting functional adaptations to diverse environmental conditions.

### Divergence time estimation

**Fig. 6:**
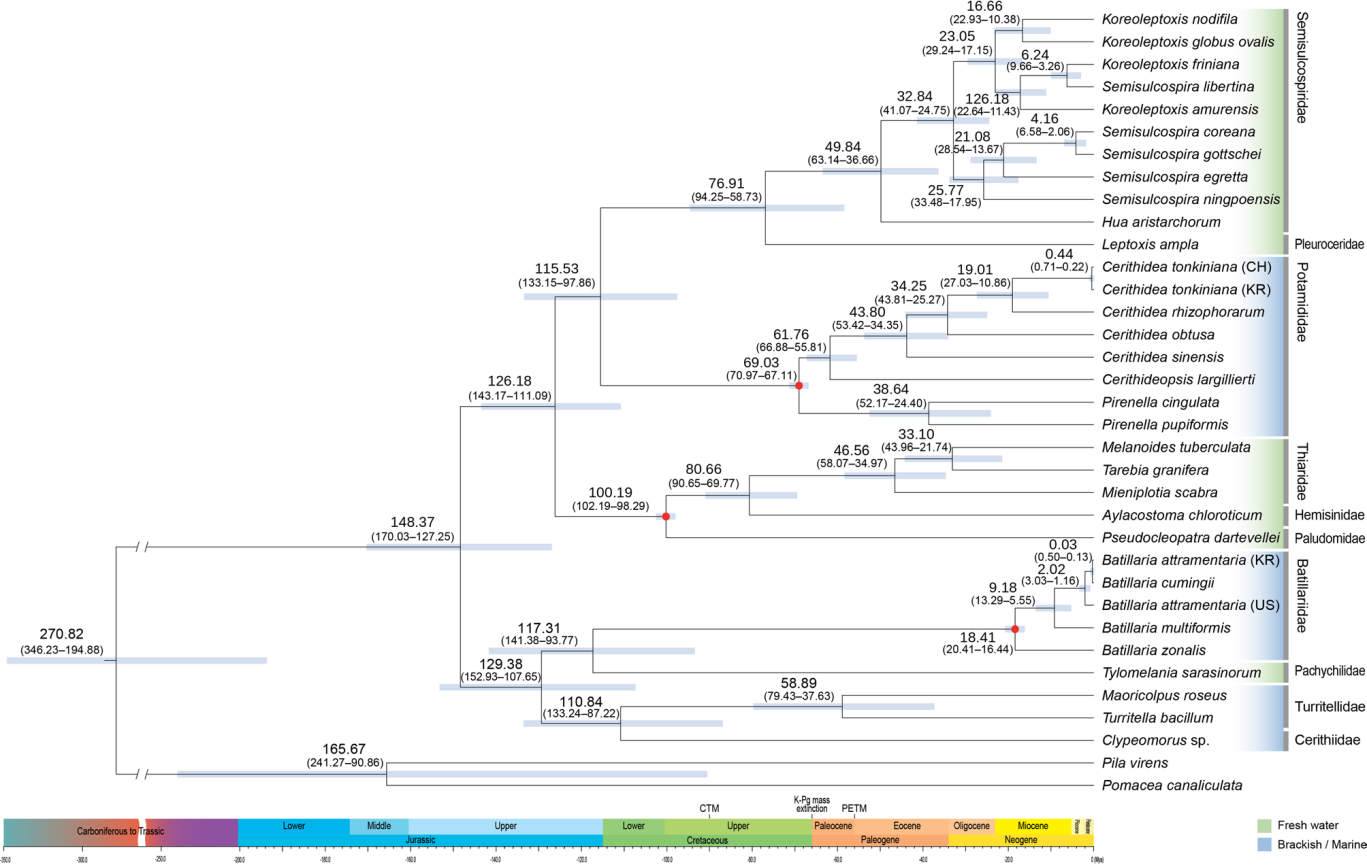
Time-calibrated phylogeny of Cerithioidea based on 13 mitochondrial PCGs. Divergence times were estimated using BEAST under a relaxed molecular clock with a Yule speciation prior. Fossil calibration points (red dots) were applied to constrain key nodes. Tip labels are color-coded by habitat (freshwater vs. brackish/marine) to illustrate ecological breadth. Node bars indicate 95% highest posterior density (HPD) intervals; mean divergence times (Ma) are shown above each node.

Divergence time analyses suggest that Ampullarioidea (Architaenioglossa) and Cerithioidea diverged before Jurassic (346.23–194.88 million years ago). Within Cerithioidea, two principal lineages subsequently separated during the Middle Jurassic to Early Cretaceous (170.03–127.25 million years ago): one lineage comprising Turritellidae, Cerithiidae, Pachychilidae, and Batillariidae, and the other including Semisulcospiridae, Pleuroceridae, Potamididae, Thiaridae, Hemisinidae, and Paludomidae. Most family-level divergences occurred in the Middle Cretaceous, whereas genus- and species-level diversification peaked during the Upper Cretaceous to Paleocene. The genus *Batillaria* experienced rapid diversification following the Miocene (Fig. 6).

## Discussion

Our sequencing of six complete mitochondrial genomes—two Batillariidae (*Batillaria multiformis* and *B. attramentaria*) and four Potamididae (*Cerithidea tonkiniana*, *C. rhizophorarum*, *Cerithideopsis largillierti*, and *Pirenella cingulata*)—revealed a high A + T content (62.51–65.56%), consistent with typical molluscan mitogenomes^23^. Among the PCGs, two overlapping regions were consistently observed: a short (7 bp) overlap between *nad4l* and *nad4*, commonly reported in Caenogastropoda, and a longer overlap (38–47 bp) between *cytb* and *nad6*^8^. These overlaps may reflect selective pressures favoring compact mitochondrial genomes by minimizing intergenic regions, which are subject to higher mutation rates^10,18^. RSCU analyses revealed a strong preference for A or T at the third codon position, consistent with the overall A + T bias observed in cerithioidean mitogenomes. Notably, although the clustering pattern of codon usage did not fully align with the phylogenetic relationships, freshwater and marine/brackish species tended to form separate groups, with the exception of *T. sarasinorum*. Although this trend has not previously been reported in gastropods, a similar pattern has been observed in ciliates, where variation in codon usage appears to correlate with habitat salinity^20^. Such patterns may arise from selection for translational efficiency or adaptations to differing metabolic demands^24^. Although codon usage bias reflects the interplay of multiple complex factors, considerable evidence suggests that both evolutionary history and ecological conditions, such as habitat, play critical roles^24,25^.

The newly sequenced mitochondrial genomes of *Batillaria attramentaria* and *Cerithidea tonkiniana* from Korea provide valuable genomic data for populations previously characterized using specimens collected elsewhere. The mitochondrial genome of *B. attramentaria* exhibited approximately 2.4% sequence divergence from that of the invasive population introduced to the United States in the 1930s^26^. This divergence likely reflects population-level genetic differentiation that accumulated following geographic and ecological isolation, rather than adaptive divergence. Nonetheless, such differentiation provides a useful baseline for future studies investigating potential adaptive evolution between native and invasive lineages^27^. Interestingly, the Korean *B. attramentaria* mitogenome was more than 99% identical to that of *B. cumingii* (MT323103) from China. Although these are currently recognized as separate species, their close genetic similarity suggests they may be conspecific, consistent with earlier hypotheses. Resolving this taxonomic question will require integrative morphological and molecular analyses using reliably identified voucher specimens. Similarly, our newly generated mitogenome of *C. tonkiniana* showed 99.7% identity with a Chinese specimen (MZ168697), with most differences confined to the putative control region (CR).

Molluscan mitochondrial genomes are well known for frequent gene rearrangements, particularly in the positions and transcriptional orientations of tRNA genes^18^. Among these changes, tRNA gene rearrangements are the most common and may play a critical role in shaping mitochondrial genome organization, as their secondary structures can function as transcriptional barriers and RNA cleavage signals^28^. The duplication–random loss model (DRLM) provides a plausible mechanism for these shifts: duplicated tRNAs may be selectively retained or lost, resulting in new gene orders over time^29,30^. The differences in tRNA arrangements observed within Cerithioidea may reflect such events. Although all analyzed cerithioidean species shared the same order of PCGs, variations in the location and strand orientation of several tRNAs, such as *trnQ*, *trnR*, *trnE*, and *trnY*, indicate lineage-specific translocations (Fig. 3). These rearrangements likely resulted from accumulated duplication–loss events, which may have occurred early in cerithioidean evolution, as they coincide with the earliest divergence points in the phylogeny (Fig. 3). However, the limited availability of complete mitogenomes complicates efforts to reconstruct their evolutionary trajectories. More extensive taxon sampling will be necessary to fully elucidate the mechanisms and evolutionary significance of mitochondrial genome rearrangements in Caenogastropoda.

We reconstructed phylogenetic trees using ML and BI methods based on three datasets, and all trees robustly supported the monophyly of Cerithioidea and consistently resolved it into two distinct lineages. Despite recent advances in cerithioidean phylogenetics, family-level relationships remain unresolved. Previous mitogenome-based studies have reported conflicting topologies: while Ling et al.^12^, Yang and Deng^31^, and Yin et al.^32^ recovered Potamididae and Semisulcospiridae as paraphyletic, other studies by Kato et al.^11^, Xu et al.^19,33^, and Forestello et al.^7^ inferred them to be monophyletic. The most recent analysis by Forestello et al.^7^, which used partial markers (16S and 28S rRNA), clustered Potamididae with Thiaridae, Hemisinidae, and Paludomidae, while excluding Semisulcospiridae. Similarly, although our results suggested that Semisulcospiridae is the sister group to Potamididae, this node received weak support across all three datasets, indicating that additional data may alter this interpretation. Additional mitogenomic data from closely related families are needed to definitively resolve this relationship. Our findings also indicate that Batillariidae formed a monophyletic group with Pachychilidae, in contrast to previous studies that grouped Batillariidae with (*Turritellidae* + *Pachychilidae*). This shift may be partially attributed to the inclusion of a new Cerithiidae sequence (*Clypeomorus* sp., PQ310514). The topology aligns with that of Forestello et al.^7^, who also recovered (*Pachychilidae* + *Batillariidae*) as sister to (*Turritellidae* + *Cerithiidae*) using 16S and 28S rRNA. Nevertheless, the weak support values and unstable branching patterns suggest that relationships among these families remain unresolved and warrant further investigation using broader taxon sampling.

Although *atp8* did not exhibit strong evidence of positive selection across all taxa, Ka/Ks analysis revealed ω > 1 in several lineages, suggesting possible episodic adaptive evolution (Fig. 4). Ka/Ks ratios > 1 indicate a suggestion of positive selection; however, they are not definitive, especially under purifying selection on synonymous sites^34^. Although codon-level methods such as FUBAR and MEME did not detect statistically significant sites in *atp8*, the combination of gene-level and branch-specific signals suggests that *atp8* may be subject to lineage-specific selective pressures. As *atp8* contributes to the assembly of the F₀ region of ATP synthase and plays a role in ATP production^35^, adaptive changes in this gene could enhance metabolic efficiency in response to diverse ecological stresses^36^. Previous studies on vertebrates have shown that *atp8* may undergo positive selection in species exposed to high-altitude, hypoxic, or extreme-temperature environments^37,38^, underscoring its potential ecological and evolutionary significance. However, given its short length, site-level analyses of *atp8* may lack statistical power, warranting careful interpretation. By contrast, *nad6* generally exhibited Ka/Ks < 1, indicating strong purifying selection (Fig. 4). *nad6* is a critical component of mitochondrial complex I, mediating electron transfer from NADH to ubiquinone during oxidative phosphorylation^39^. Because it spans the inner mitochondrial membrane, *nad6* is believed to facilitate structural rearrangements essential for proton pumping. Positive selection on *nad6* has previously been associated with local adaptation in oxidative phosphorylation, potentially driving energy production under distinct environmental conditions^40,41^. Despite the overall evidence of purifying selection, both FUBAR and MEME identified three codons in *nad6* under positive selection, suggesting that certain sites may experience episodic or lineage-specific adaptive pressures^42,43^.

While only one overlap was observed between the sites identified by the site model and those detected by the branch-site model, this discrepancy is not unexpected. The site model detects pervasive selection across the entire phylogeny, whereas the branch-site model focuses on episodic selection occurring in specific lineages. MEME identified a codon overlapping with Branch #3, supporting the hypothesis that episodic selection may have contributed to the divergence of major cerithioidean lineages. The detection of positively selected codons along Branches #1–#3 suggests that substitutions at these evolutionary nodes may have facilitated key functional shifts or ecological adaptations. Site-level methods (FUBAR, MEME) and branch-specific approaches (BEB in CodeML) identified distinct sets of positively selected codons, indicating the co-occurrence of both pervasive and episodic selection across mitochondrial genes. *nad6* consistently exhibited strong signals across multiple methods, highlighting its potential role in lineage-specific adaptation within Cerithioidea. AlphaFold-based structural modeling of *nad6* and related proteins revealed that α-helices and transmembrane domains are conserved across the superfamily, underscoring their critical role in oxidative phosphorylation. However, notable variation in predicted disordered regions, particularly near the C-terminal ends, suggests that adaptive changes may accumulate in more structurally flexible regions. These variations could enable regulatory modifications without disrupting key structural motifs^44,45^. Notably, the three observed size variants of *nad6* (507, 543, and 552 bp) corresponded with distinct taxonomic clusters, implying that structural differences in disordered regions may contribute to lineage-specific adaptations to environmental factors such as temperature or salinity^46^. Recent research increasingly supports the role of adaptive evolution in mitochondrial PCGs among marine and brackish invertebrates, likely driven by fluctuations in temperature, salinity, and oxygen levels^14,16^. Despite differing levels of functional constraint, both *atp8* and *nad6* can exhibit episodes of positive selection at the gene or codon level, optimizing energy metabolism in response to dynamic conditions^37^. Analyses of site- and region-specific adaptations offer valuable insights into how invertebrate populations mitigate environmental stress, highlighting the pivotal role of mitochondrial function in ecological and evolutionary resilience.

Although Cerithioidea has a rich fossil record, accurately classifying fossil species remains challenging. To address this, we established calibration points based on a recent revision of fossil taxa^46^. Our estimates place the divergence of Semisulcospiridae at approximately 77.65 million years ago (95.19–60.10 Ma), which is older than the approximately 44.5 Ma range (66–24 Ma) reported by Neiber et al.^48^. Strong and Köhler^49^ proposed that this clade began diversifying around 55 Ma, with the most recent common ancestor of *Semisulcospira* emerging approximately 25 Ma. Our estimate for that event, 33.07 Ma (41.24–24.89 Ma), is broadly consistent with both this previous finding and Japanese fossil records dated to approximately 23–15 Ma^50^. Reid et al.^47^ documented Cerithideopsis fossils in the Middle to Late Eocene, whereas our analyses infer an earlier, Paleocene origin. For Batillariidae, we placed the genus *Batillaria* in the Early Miocene, considerably more recent than fossil-based estimates suggesting a Late Paleocene to Eocene origin^51^. Hazhauser et al. (2023) noted that Potamididae and Batillariidae were highly diverse during the Miocene Climate Optimum, but subsequently experienced the extinction of many large-bodied species^52^. This extinction event may partly explain discrepancies between molecular divergence times and the fossil record. While Han et al.^53^ inferred the origin of Cerithioidea around 150 Ma based on whole-genome data, our mitochondrial estimate suggests a much deeper origin, possibly extending back to the Cambrian and Early Jurassic intervals (274.29 million years ago; 351.07–197.51 Ma). Divergence time differences may result from variations in molecular clock calibrations, gene regions analyzed, taxon sampling, and analytical models. Nonetheless, our study, based on mitogenomic data, produced relatively narrow confidence intervals. Future efforts integrating broader taxon sampling, improved calibration points, and more comprehensive datasets will help further refine these estimates.

An intriguing finding of our study is that the divergence of major cerithioidean families occupying different habitats occurred primarily during the Cretaceous Thermal Maximum (CTM; ~ 90 Ma), with further increases in lineage diversity inferred around the Paleocene–Eocene Thermal Maximum (PETM; ~ 56 Ma), both periods characterized by elevated global temperatures^54,55^. BEAST analyses suggest that the earliest family-level divergences coincided with the CTM, a time of high atmospheric CO_2_ concentrations, extremely warm equatorial sea surface temperatures, and widespread oceanic anoxia. These extreme conditions likely weakened geographic and ecological barriers, such as salinity gradients and thermal thresholds, facilitating dispersal and early diversification. In addition, our results indicate that cerithioidean lineages surviving the K–Pg mass extinction (~ 66 Ma) subsequently underwent increased diversification, suggesting that ecological opportunities created by this global crisis were rapidly exploited^56^. During the PETM, further warming, sea-level rise, and shifts in ocean chemistry appear to have promoted continued radiation, broadening lineage richness and ecological breadth^57^. Taken together, these events highlight how repeated episodes of climatic extremes and biotic crises shaped the tempo and mode of cerithioid evolution.

Overall, our findings underscore the interplay between mitochondrial genome evolution and global environmental fluctuations in shaping the diversification of Cerithioidea. Continued research that integrates whole-genome sequencing, paleoenvironmental modeling, and comprehensive taxon sampling will be essential for fully reconstructing the evolutionary trajectory of Cerithioidea and understanding how historical climate shifts influenced lineage-specific adaptations.

## Materials and methods

### Sample collection, DNA extraction, and sequencing

Specimens were collected from intertidal zones at different sites along the western and southern coasts of South Korea (Supplementary Table S1). The species used in this study are not subject to animal ethics regulations in South Korea or internationally. Thus, no ethical approval was required, but all efforts were made to minimize environmental impact and animal stress. Species identification was based on morphological features and confirmed through analysis of partial sequences from a molecular marker. Total genomic DNA was extracted from a section of muscular foot tissue using a DNeasy Blood and Tissue kit (Qiagen, Germany). DNA quality and quantity were assessed using a NanoDrop 4000 spectrophotometer (Thermo Fisher Scientific, USA). A fragment of the mitochondrial *cox1* gene was amplified using the primers LCO1490 and HCO2198^58^. PCR amplification was performed under the following conditions: initial denaturation at 94 °C for 10 min, followed by 35 cycles of denaturation at 94 °C for 30 s, primer annealing at 48 °C for 1 min, and extension at 72 °C for 1 min, and a final extension at 72 °C for 10 min. PCR products were purified using the QIAquick PCR Purification kit (QIAGEN Co., USA) and sequenced directly using an ABI Prism 3730 DNA sequencer (PerkinElmer Inc., USA) with the BigDye Terminator Sequencing kit (PerkinElmer Inc., USA). After verifying the reliability of the nucleotide sequences, the confirmed *cox1* sequences were subjected to BLAST searches. Complete mitochondrial genomes were sequenced using next-generation sequencing (NGS) technology. Sequencing libraries were constructed with an average insert size of 150 bp using the QIAseq FX single cell DNA library kit (Qiagen, Germany). Sequencing was performed using the Illumina HiSeq 4000 platform. Raw sequencing reads were subjected to quality control prior to assembly. Adapter sequences and low-quality bases were trimmed, and reads with a Phred quality score below Q30 were removed. Only high-quality reads passing these thresholds were retained for downstream analyses.

### Mitogenome assembly, annotation, and characterization

The mitochondrial genomes of six cerithioidean species were assembled using NOVOPlasty v4.3.1^59^, with partial sequences of the *cox1* gene used as seed sequences. PCGs were identified using ORF Finder (https://www.ncbi.nlm.nih.gov/orffinder) and MITOS^60^. A total of 22 tRNA genes were predicted using tRNAscan-SE (https://lowelab.ucsc.edu/tRNAscan-SE/) and ARWEN^61^. rRNA genes were annotated through comparative analyses with other caenogastropod mitogenomes, and their boundaries were manually adjusted based on the positions of adjacent genes^62^. To visualize genome structure, each mitochondrial genome was illustrated in circular format using the Proksee web server (https://proksee.ca/) (Fig. 1). The complete mitochondrial genome sequences and their corresponding annotation files for all six species were uploaded from GenBank (Supplementary Table S1).

Following annotation, nucleotide composition and RSCU for the PCGs were analyzed using the CAIcal server (http://genomes.urv.es/CAIcal/). Nucleotide skewness was calculated using the following formulas: AT skew = (A − T)/(A + T) and GC skew = (G − C)/(G + C)^63^. To assess codon usage patterns across species, RSCU values were computed for each codon in each species. The resulting RSCU matrix was visualized as a heatmap, with codons on the x-axis and species on the y-axis. To highlight similarities in codon usage patterns, hierarchical clustering of species was performed using Ward’s minimum variance method (ward.D2) and Euclidean distance, and the clustering dendrograms were displayed alongside the heatmap. Stop codons (TAG and TAA) were excluded to minimize the effect of their amino acid composition. All analyses were conducted in R v.4.5.1^64^ using the *pheatmap* package^65^. To evaluate the robustness of the species clusters, multiscale bootstrap resampling was performed using the *pvclust* package^66^. Clusters with approximately unbiased (AU) *p*-values > 95% were considered statistically significant and are marked with red dots on the dendrogram (Fig. 2).

### Phylogenetic analyses

Phylogenetic trees were constructed using ML and BI methods based on sequences from 13 PCGs and two rRNA genes from 33 cerithioidean species. Two additional caenogastropods were included as outgroups (Fig. 3, Supplementary Fig. S7). The PCG sequences were aligned, and start and stop codons were trimmed using BioEdit v7.2^67^. Ambiguously aligned regions in the rRNA genes were removed using Gblocks v0.91b with default parameters^68^. Three datasets were used for the analysis: (1) a combined nucleotide dataset comprising 13 PCGs and two rRNAs, (2) a nucleotide dataset of the 13 PCGs, and (3) amino acids dataset. Partitioning schemes were determined using ModelFinder v2.2.0^69^, which selected the best-fit models for each gene partition based on the Bayesian information criterion (BIC). The selected models were TIM + F + I + G4 for *cox1*, GTR + F + I + G4 for *cox2* + *nad4l* + *nad3*, TVM + F + I + G4 for *nad4* + *nad5* + *cytb*, GTR + F + I + G4 for *cox3*, TVM + F + I + G4 for *nad6* + *atp8*, TVM + F + I + G4 for *nad1* + *atp6*, TVM + F + I + G4 for *nad2*, and TVM + F + I + G4 for *rrnS* + *rrnL* for the nucleotide datasets, and mtVer + F + I + G4 for the amino acid dataset. ML analyses were performed using the IQ-TREE web server (http://iqtree.cibiv.univie.ac.at/) with 10,000 ultrafast bootstrap replicates. BI analyses were conducted using MrBayes v3.2.7a^70^, using the previously identified best-fit substitution models. Two simultaneous Monte Carlo Markov Chain (MCMC) runs were executed for 10,000,000 generations, with trees sampled every 1,000 generations. The first 25% of sampled trees were discarded as burn-in. Convergence of the independent MCMC runs was assessed by examining the mean standard deviation of split frequencies (< 0.01). The resulting phylogenetic trees were visualized using FigTree v1.4.4^71^.

### Selection analyses

The strength of selection against nonsynonymous substitutions relative to synonymous substitutions was assessed by calculating the ratio of nonsynonymous substitutions per nonsynonymous site (Ka) to synonymous substitutions per synonymous site (Ks). To compare patterns of sequence evolution among cerithioidean species, Ka/Ks ratios were calculated from pairwise DNA sequence comparisons using the *yn00* module in PAML v4.8^72^ (Fig. 4).

Codons potentially under selection were identified using the HyPhy package via the DataMonkey web server (https://www.datamonkey.org/). The fast unconstrained Bayesian approximation (FUBAR) method was used to detect codons undergoing pervasive purifying or diversifying selection^73^, and the mixed effects model of evolution (MEME) was used to identify episodic positive selection^22^. Additionally, an adaptive branch-site random effects likelihood (aBSREL) was used to detect episodic selection across all branches^74^. Statistical significance was determined using a posterior probability (PP) > 0.9 for FUBAR, and *p*-values < 0.05 for MEME and aBSREL. To further test for positive selection at specific codons or lineages, site models implemented in CodeML within the PAML package were also applied. Six different site models were tested using equal codon frequencies, as proposed by Yang et al.^75^: M0 (one ω ratio), M1a (nearly neutral), M2a (positive selection), M3 (discrete), M7 (beta), and M8 (beta and ω). Likelihood ratio tests (LRTs) were conducted to compare null models with those that allow ω > 1, as follows: M0 vs. M1a to test for a single ω ratio across codons, M0 vs. M3 to assess variable selection pressure among sites, and M1a vs. M2a and M7 vs. M8 to detect evidence of positive selection. When LRT results supported positive selection, Bayes empirical Bayes (BEB) analysis was used to estimate the PP of codons under positive selection (ω > 1) in models M2a and M8. Codons with PP > 0.95 were considered to be under positive selection^75^ (Table 2). Additionally, branches identified by aBSREL as undergoing episodic diversifying selection (*p* < 0.05) were designated as foreground branches for subsequent branch-site model analyses using CodeML. For these branches, the M2a branch-site model was applied to detect codon-specific positive selection. Sites with BEB posterior probabilities exceeding 0.95 were considered to be under positive selection within those lineages (Table 2).

To assess whether codons or genes under positive selection may lead to structural or functional changes in the encoded proteins, the AlphaFold Server (https://alphafoldserver.com/) was used to generate three-dimensional structure predictions (Fig. 5). The resulting models were examined to identify potential alterations in regions such as binding sites, transmembrane domains, or disordered regions.

### Divergence time estimation

Divergence time estimation was conducted using BEAST v2.6.0^76^ based on concatenated sequences of 13 PCGs. Fossil calibration points were incorporated using BEAUti v2 to generate the XML file. Three fossil calibration points were applied based on the fossil records: (1) the crown group of Thiaridae + Hemisinidae + Paludomidae was constrained to the Mid-Cretaceous (100.5 Ma)^77^, (2) the earliest known fossil of the family Potamididae from the Late Cretaceous (66.0–72.1 Ma)^47^, and (3) a fossil of the genus *Batillaria* from the Early Miocene (15.97–23.03 Ma)^51^. Fossil calibration priors were implemented as log-normal distributions, following standard practice to accommodate uncertainty in fossil ages. For each calibration, the offset was set to the minimum bound of the corresponding fossil age range, while the mean and standard deviation in log space were both set to 1.0, producing a moderately right-skewed distribution. Accordingly, the offset values were 100.5 Ma for the Thiaridae + Hemisinidae + Paludomidae calibration, 66.0 Ma for Potamididae, and 15.97 Ma for *Batillaria*. Divergence times were estimated under a Yule speciation prior with a relaxed log-normal molecular clock. The best-fitting substitution model for each partition was applied in BEAST. Bayesian inference was performed using 20 million MCMC generations, with parameters sampled every 1,000 generations. The initial 25% of samples were discarded as burn-in. Two independent runs with different random seeds were performed to ensure convergence. Tracer v1.7^78^ was used to assess convergence and mixing of parameters, confirming that all major effective sample sizes (ESS) exceeded 200 after burn-in. The log and tree files from independent runs were combined using LogCombiner, and the resulting posterior distribution of trees was summarized as a maximum clade credibility (MCC) tree in TreeAnnotator v2.6.0. The final tree was visualized and edited in FigTree v1.4.4^71^ (Fig. 6).

## Supplementary Information

Supplementary Information.

## Data Availability

The complete mitochondrial genome sequence generated and analyzed during the current study has been deposited in the NCBI GenBank database under the accession numbers PV619093–PV619098. All other relevant data are available from the corresponding author upon reasonable request.

## References

[1] MolluscaBase. *MolluscaBase*. https://www.molluscabase.org (2025). Accessed 2 April 2025.

[2] Strong, E. E. et al. Phylogeny of the gastropod superfamily Cerithioidea using morphology and molecules. *Zool. J. Linn. Soc.* **162**, 43–89. 10.1111/j.1096-3642.2010.00670.x (2011).

[3] Ponder, W. F. et al. Caenogastropod phylogeny. In *Molluscan Phylogeny* (eds Ponder, W. F. & Lindberg, D. R.) 331–383 (University of California Press, 2008).

[4] Houbrick, R. S. Cerithiodean phylogeny. In *Prosobranch Phylogeny* (ed. Ponder, W. F.) *Malacol. Rev. Suppl.* **4**, 88–128 (1988).

[5] Simone, L. R. L. Phylogenetic analysis of Cerithioidea (Mollusca: Caenogastropoda) based on comparative morphology. *Arq. Zool.* **36**, 147–263. 10.11606/issn.2176-7793.v36i2p147-263 (2001).

[6] Lydeard, C. et al. Molecular phylogeny of a circum-global, diverse gastropod superfamily (Cerithioidea: Mollusca: Caenogastropoda): pushing the deepest phylogenetic limits of mitochondrial LSU rDNA sequences. *Mol. Phylogenet. Evol.* **22**, 399–406. 10.1006/mpev.2001.1072 (2002). 11884164 10.1006/mpev.2001.1072

[7] Forestello, E. et al. The mitochondrial genome of the threatened freshwater snail Aylacostoma chloroticum (Gastropoda, Hemisinidae) from the High Paraná River and the phylogenetic relationships of Cerithioidea. *Zoosyst. Evol.* **101**, 353–368. 10.3897/zse.101.142841 (2025).

[8] Choi, E. H., Choi, N. R. & Hwang, U. W. The mitochondrial genome of an endangered freshwater snail *Koreoleptoxis nodifila* (Caenogastropoda: Semisulcospiridae) from South Korea. *Mitochondrial DNA B* **6**, 1120–1123. 10.1080/23802359.2021.1901626 (2021). 10.1080/23802359.2021.1901626 PMC7995809 33796761

[9] Lee, Y., Kim, K. B., Choi, E. H. & Hwang, U. W. Complete mitochondrial genome of the worm snail *Thylacodes adamsii* (Littorinimorpha: Vermetidae) from South Korea. *Mitochondrial DNA B* **9**, 753–757. 10.1080/23802359.2024.2368209 (2024). 10.1080/23802359.2024.2368209 PMC11185085 38895513

[10] Boore, J. L. Animal mitochondrial genomes. *Nucleic Acids Res.* **27**, 1767–1780 (1999). 10101183 10.1093/nar/27.8.1767 PMC148383

[11] Kato, S. et al. The mitochondrial genome of the threatened tideland snail *Pirenella pupiformis* (Mollusca: Caenogastropoda: Potamididae) determined by shotgun sequencing. *Mitochondrial DNA B* **7**, 632–634. 10.1080/23802359.2022.2060143 (2022). 10.1080/23802359.2022.2060143 PMC9004528 35425860

[12] Ling, Y. et al. The complete mitochondrial genome of *Melanoides tuberculata* (Müller, 1774) in Guangdong, China. *Mitochondrial DNA B* **7**, 1319–1320. 10.1080/23802359.2022.2054735 (2022). 10.1080/23802359.2022.2054735 PMC9310916 35898661

[13] Weersing, K. & Toonen, R. J. Population genetics, larval dispersal, and connectivity in marine systems. *Mar. Ecol. Prog. Ser.* **393**, 1–12 (2009).

[14] Sun, J. et al. Adaptation to deep-sea chemosynthetic environments as revealed by mussel genomes. *Nat. Ecol. Evol.* **1**, 0121 (2017). 10.1038/s41559-017-0121 28812709

[15] Tomanek, L. Environmental proteomics of the blue mussel *Mytilus*: implications for thermal stress and acclimation. *Mar. Biotechnol.* **14**, 106–115 (2012).

[16] Zouros, E. The exceptional mitochondrial DNA system of the mussel family Mytilidae. *Genes Genet. Syst.* **75**, 313–318 (2000). 11280005 10.1266/ggs.75.313

[17] Grande, C., Templado, J. & Zardoya, R. Evolution of gastropod mitochondrial genome arrangements. *BMC Evol. Biol.* **8**, 61. 10.1186/1471-2148-8-61 (2008). 18302768 10.1186/1471-2148-8-61 PMC2291457

[18] Ghiselli, F. et al. Molluscan mitochondrial genomes break the rules. *Philos. Trans. R. Soc. Lond. B Biol. Sci.* **376**, 20200159. 10.1098/rstb.2020.0159 (2021). 10.1098/rstb.2020.0159 PMC8059616 33813887

[19] Xu, Y. et al. The mitochondrial genome of *Hua aristarchorum* (Heude, 1889) (Gastropoda, Cerithioidea, Semisulcospiridae) and its phylogenetic implications. *ZooKeys* **1192**, 237–255. 10.3897/zookeys.1192.116269 (2024). 38433759 10.3897/zookeys.1192.116269 PMC10905624

[20] Huang, N. et al. Molecular evolutionary analyses of Euplotes species living in freshwater and marine habitats: A mitogenomic perspective. *Front. Mar. Sci.* **8**, 627879. 10.3389/fmars.2021.627879 (2021).

[21] Yang, Z. & Bielawski, J. P. Statistical methods for detecting molecular adaptation. *Trends Ecol. Evol.* **15**, 496–503 (2000). 11114436 10.1016/S0169-5347(00)01994-7 PMC7134603

[22] Murrell, B. et al. Detecting individual sites subject to episodic diversifying selection. *PLoS Genet.* **8**, e1002764. 10.1371/journal.pgen.1002764 (2012). 22807683 10.1371/journal.pgen.1002764 PMC3395634

[23] Sun, S. E., Li, Q., Kong, L. & Yu, H. Multiple reversals of strand asymmetry in molluscs mitochondrial genomes, and consequences for phylogenetic inferences. *Mol. Phylogenet. Evol.* **118**, 222–231 (2018). 29038046 10.1016/j.ympev.2017.10.009

[24] Behura, S. K. & Severson, D. W. Codon usage bias: Causative factors, quantification methods and genome-wide patterns: With emphasis on insect genomes. *Biol. Rev.* **88**, 49–61 (2013). 22889422 10.1111/j.1469-185X.2012.00242.x

[25] Goodarzi, H. et al. Amino acid and codon usage profiles: adaptive changes in the frequency of amino acids and codons. *Gene* **407**, 30–41 (2008). 17977670 10.1016/j.gene.2007.09.020

[26] Wasson, K. et al. Biological invasions of estuaries without international shipping: the importance of intraregional transport. *Biol. Conserv.* **102**, 143–153 (2001).

[27] Prentis, P. J. et al. Adaptive evolution in invasive species. *Trends Plant Sci.* **13**, 288–294. 10.1016/j.tplants.2008.03.004 (2008). 18467157 10.1016/j.tplants.2008.03.004

[28] Fernández-Silva, P., Enriquez, J. A. & Montoya, J. Replication and transcription of mammalian mitochondrial DNA. *Exp. Physiol.* **88**, 41–56 (2003). 12525854 10.1113/eph8802514

[29] Cantatore, P. et al. Duplication and remoulding of tRNA genes during the evolutionary rearrangement of mitochondrial genomes. *Nature* **329**, 853–855. 10.1038/329853a0 (1987). 3670390 10.1038/329853a0

[30] Rawlings, T. A., MacInnis, M. J., Bieler, R., Boore, J. L. & Collins, T. M. Sessile snails, dynamic genomes: gene rearrangements within the mitochondrial genome of a family of caenogastropod molluscs. *BMC Genomics* **11**, 440. 10.1186/1471-2164-11-440 (2010). 20642828 10.1186/1471-2164-11-440 PMC3091637

[31] Yang, S. & Deng, Z. The complete mitochondrial genome of *Cerithidea tonkiniana* (Mabille, 1887) in Guangxi, China. *Mitochondrial DNA B* **7**, 669–670. 10.1080/23802359.2021.2006813 (2022). 10.1080/23802359.2021.2006813 PMC9037160 35478855

[32] Yin, N. et al. Complete mitochondrial genome of the freshwater snail *Tarebia granifera* (Lamarck, 1816). *Mitochondrial DNA B* **7**, 259–261. 10.1080/23802359.2022.2026832 (2022). 10.1080/23802359.2022.2026832 PMC8788337 35087949

[33] Xu, Y. B. et al. A new species of *Semisulcospira* O. Boettger, 1886 from Fujian, China with mitochondrial genome and its phylogenetic implications. *Zoosyst. Evol.* **101**, 17–34. 10.3897/zse.101.136882(2025).

[34] Spielman, S. J. & Wilke, C. O. The relationship between dN/dS and scaled selection coefficients. *Mol. Biol. Evol.* **32**, 1097–1108. 10.1093/molbev/msv003 (2015). 25576365 10.1093/molbev/msv003 PMC4379412

[35] Jonckheere, A. I., Smeitink, J. A. & Rodenburg, R. J. Mitochondrial ATP synthase: architecture, function and pathology. *J. Inherit. Metab. Dis.* **35**, 211–225. 10.1007/s10545-011-9382-9 (2012). 21874297 10.1007/s10545-011-9382-9 PMC3278611

[36] Ramos, N. I. et al. Selection in coral mitogenomes, with insights into adaptations in the deep sea. *Sci. Rep.* **13**, 6016. 10.1038/s41598-023-31243-1 (2023). 37045882 10.1038/s41598-023-31243-1 PMC10097804

[37] Ramos, B. et al. Landscape genomics: natural selection drives the evolution of mitogenome in penguins. *BMC Genom.* **19**, 53. 10.1186/s12864-017-4424-9 (2018). 10.1186/s12864-017-4424-9 PMC5771141 29338715

[38] Priyono, D. S. et al. The first complete mitochondrial genome of Sumatran striped rabbit *Nesolagus netscheri* (Schlegel, 1880), and its phylogenetic relationship with other Leporidae. *Sci. Rep.* **15**, 2002. 10.1038/s41598-025-85212-x (2025). 39814825 10.1038/s41598-025-85212-x PMC11735860

[39] Bai, Y. & Attardi, G. The mtDNA-encoded ND6 subunit of mitochondrial NADH dehydrogenase is essential for the assembly of the membrane arm and the respiratory function of the enzyme. *EMBO J.* **17**, 4848–4858 (1998). 9707444 10.1093/emboj/17.16.4848 PMC1170814

[40] Ning, T., Xiao, H., Li, J., Hua, S. & Zhang, Y. P. Adaptive evolution of the mitochondrial ND6 gene in the domestic horse. *Genet. Mol. Res.* **9**, 144–150 (2010). 20198570 10.4238/vol9-1gmr705

[41] Stefanović, M. et al. Positive selection and precipitation effects on the mitochondrial NADH dehydrogenase subunit 6 gene in brown hares (*Lepus europaeus*) under a phylogeographic perspective. *PLoS ONE* **14**, e0224902. 10.1371/journal.pone.0224902 (2019). 31703111 10.1371/journal.pone.0224902 PMC6839855

[42] Echave, J., Spielman, S. & Wilke, C. Causes of evolutionary rate variation among protein sites. *Nat. Rev. Genet.* **17**, 109–121. 10.1038/nrg.2015.18 (2016). 26781812 10.1038/nrg.2015.18 PMC4724262

[43] Dhar, D., Dey, D., Basu, S. & Fortunato, H. Insight into the adaptive evolution of mitochondrial genomes in intertidal chitons. *J. Molluscan Stud.* **87**, eyab018. 10.1093/mollus/eyab018(2021).

[44] Liu, J., Faeder, J. R. & Camacho, C. J. Toward a quantitative theory of intrinsically disordered proteins and their function. *Proc. Natl Acad. Sci. USA* **106**, 19819–19823. 10.1073/pnas.0907710106 (2009). 19903882 10.1073/pnas.0907710106 PMC2775701

[45] Forman-Kay, J. D. & Mittag, T. From sequence and forces to structure, function, and evolution of intrinsically disordered proteins. *Structure* **21**, 1492–1499. 10.1016/j.str.2013.08.001 (2013). 24010708 10.1016/j.str.2013.08.001 PMC4704097

[46] Vermeij, G. J. *A natural history of shells*. (Princeton University Press, 1993).

[47] Reid, D. G., Dyal, P., Lozouet, P., Glaubrecht, M. & Williams, S. T. Mudwhelks and mangroves: the evolutionary history of an ecological association (Gastropoda: Potamididae). *Mol. Phylogenet. Evol.* **47**, 680–699. 10.1016/j.ympev.2008.01.003 (2008). 18359643 10.1016/j.ympev.2008.01.003

[48] Neiber, M. T. & Glaubrecht, M. Unparalleled disjunction or unexpected relationships? Molecular phylogeny and biogeography of Melanopsidae (Caenogastropoda: Cerithioidea), with the description of a new family and a new genus from the ancient continent Zealandia. *Cladistics* **35**, 401–425. 10.1111/cla.12361 (2019). 34633705 10.1111/cla.12361

[49] Strong, E. E. & Köhler, F. Morphological and molecular analysis of ‘*Melania jacqueti*’ Dautzenberg and Fischer, 1906. *Zool. Scr.* **38**, 483–502 (2009).

[50] Matsuoka, K. & Taguchi, E. A new species of *Sulcospira* (Pachychilidae: Gastropoda) from the Miocene Katsuta Group in Okayama Prefecture, Southwest Japan. *Bull. Mizunami Fossil Mus.* **39**, 55–57 (2013).

[51] Ozawa, T., Köhler, F., Reid, D. G. & Glaubrecht, M. Tethyan relicts on continental coastlines of the northwestern Pacific Ocean and Australasia: Molecular phylogeny and fossil record of batillariid gastropods (Caenogastropoda, Cerithioidea). *Zool. Scr.* **38**, 503–525. 10.1111/j.1463-6409.2009.00390.x (2009).

[52] Harzhauser, M. et al. Oligocene to Pleistocene mudwhelks (Gastropoda: Potamididae, Batillariidae) of the Eurasian Paratethys Sea – Diversity, origins and mangroves. *Palaeogeogr. Palaeoclimatol. Palaeoecol.* **630**, 111811. 10.1016/j.palaeo.2023.111811 (2023).

[53] Han, X. et al. Out of the ocean: the timescale of molluscan evolution based on phylogenomics revealed the ages of mollusks’ evolutionary transitions into the novel environment. *Front. Ecol. Evol.* **12**, 1327007. 10.3389/fevo.2024.1327007 (2024).

[54] Brain, T. H. et al. The rise and fall of the cretaceous hot greenhouse climate. *Glob. planet. change.* **167**, 1–23. 10.1016/j.gloplacha.2018.04.004 (2018).

[55] Li, M. et al. Astrochronology of the paleocene-eocene thermal maximum on the Atlantic coastal plain. *Nat. Commun.* **13**, 5618. 10.1038/s41467-022-33390-x (2022). 36153313 10.1038/s41467-022-33390-x PMC9509358

[56] Delicado, D., Hauffe, T. & Wilke, T. Fifth mass extinction event triggered the diversification of the largest family of freshwater gastropods (Caenogastropoda: Truncatelloidea: Hydrobiidae). *Cladistics* **40**, 82–96. 10.1111/cla.12558 (2024). 37712584 10.1111/cla.12558

[57] Speijer, R. et al. Response of marine ecosystems to deep-time global warming: A synthesis of biotic patterns across the Paleocene-Eocene thermal maximum (PETM). *Aust. J. Earth Sci.* **105**, 6–16 (2012).

[58] Folmer, O., Black, M., Hoeh, W., Lutz, R. & Vrijenhoek, R. DNA primers for amplification of mitochondrial cytochrome c oxidase subunit I from diverse metazoan invertebrates. *Mol. Mar. Biol. Biotechnol.* **3**, 294–299 (1994). 7881515

[59] Dierckxsens, N., Mardulyn, P. & Smits, G. NOVOPlasty: de novo assembly of organelle genomes from whole genome data. *Nucleic Acids Res.* **45**, e18. 10.1093/nar/gkw955 (2017). 28204566 10.1093/nar/gkw955 PMC5389512

[60] Bernt, M. et al. MITOS: Improved de novo metazoan mitochondrial genome annotation. *Mol. Phylogenet. Evol.* **69**, 313–319. 10.1016/j.ympev.2012.08.023 (2013). 22982435 10.1016/j.ympev.2012.08.023

[61] Laslett, D. & Canbäck, B. ARWEN, a program to detect tRNA genes in metazoan mitochondrial nucleotide sequences. *Bioinformatics* **24**, 172–175. 10.1093/bioinformatics/btm573 (2008). 18033792 10.1093/bioinformatics/btm573

[62] Boore, J. L., Macey, J. R. & Medina, M. Sequencing and comparing whole mitochondrial genomes of animals. *Methods Enzymol.* **395**, 311–348. 10.1016/S0076-6879(05)95019-2 (2005). 15865975 10.1016/S0076-6879(05)95019-2

[63] Perna, N. T. & Kocher, T. D. Patterns of nucleotide composition at fourfold degenerate sites of animal mitochondrial genomes. *J. Mol. Evol.* **41**, 353–358. 10.1007/BF01215182 (1995). 10.1007/BF00186547 7563121

[64] R Core Team. *R: A Language and Environment for Statistical Computing*. R Foundation for Statistical Computing, Vienna, Austria. https://www.R-project.org/ (2025).

[65] Kolde, R. *pheatmap: Pretty Heatmaps* version 1.0.12 from CRAN. https://rdrr.io/cran/pheatmap/ (2019).

[66] Suzuki, R. & Shimodaira, H. Pvclust: an R package for assessing the uncertainty in hierarchical clustering. *Bioinformatics* **22**, 1540–1542. 10.1093/bioinformatics/btl117 (2006). 16595560 10.1093/bioinformatics/btl117

[67] Hall, T. A. BioEdit: a user-friendly biological sequence alignment editor and analysis program for Windows 95/98/NT. *Nucl. Acids Symp. Ser.* **41**, 95–98 (1999).

[68] Castresana, J. Selection of conserved blocks from multiple alignments for their use in phylogenetic analysis. *Mol. Biol. Evol.* **17**, 540–552. 10.1093/oxfordjournals.molbev.a026334 (2000). 10742046 10.1093/oxfordjournals.molbev.a026334

[69] Kalyaanamoorthy, S. et al. ModelFinder: Fast model selection for accurate phylogenetic estimates. *Nat. Methods* **14**, 587–589. 10.1038/nmeth.4285 (2017). 28481363 10.1038/nmeth.4285 PMC5453245

[70] Ronquist, F. et al. MrBayes 3.2: Efficient Bayesian phylogenetic inference and model choice across a large model space. *Syst. Biol.* **61**, 539–542 (2012). 10.1093/sysbio/sys029 PMC3329765 22357727

[71] Rambaut, A. *Figtree ver 1.4.4*. Institute of Evolutionary Biology, University of Edinburgh (2018).

[72] Yang, Z. PAML 4: Phylogenetic analysis by maximum likelihood. *Mol. Biol. Evol.* **24**, 1586–1591 (2007). 17483113 10.1093/molbev/msm088

[73] Murrell, B. et al. FUBAR: a fast, unconstrained Bayesian approximation for inferring selection. *Mol. Biol. Evol.* **30**, 1196–1205. 10.1093/molbev/mst030 (2013). 23420840 10.1093/molbev/mst030 PMC3670733

[74] Smith, M. D. et al. Less is more: an adaptive branch-site random effects model for efficient detection of episodic diversifying selection. *Mol. Biol. Evol.* **32**, 1342–1353 (2015). 25697341 10.1093/molbev/msv022 PMC4408413

[75] Yang, Z., Nielsen, R., Goldman, N. & Pedersen, A. M. Codon-substitution models for heterogeneous selection pressure at amino acid sites. *Genetics* **155**, 431–449. 10.1093/genetics/155.1.431 (2000). 10790415 10.1093/genetics/155.1.431 PMC1461088

[76] Bouckaert, R. et al. BEAST 2: A software platform for Bayesian evolutionary analysis. *PLoS Comput. Biol.* **10**, e1003537. 10.1371/journal.pcbi.1003537 (2014). 24722319 10.1371/journal.pcbi.1003537 PMC3985171

[77] Beu, A., Marshall, B. & Reay, M. Mid-cretaceous (Albian–Cenomanian) freshwater Mollusca from the Clarence Valley, Marlborough, New Zealand, and their biogeographical significance. *Cretac. Res.* **49**, 134–151. 10.1016/j.cretres.2014.02.011 (2014).

[78] Rambaut, A., Drummond, A. J., Xie, D., Baele, G. & Suchard, M. A. Posterior summarisation in Bayesian phylogenetics using Tracer 1.7. *Syst. Biol.* syy032. 10.1093/sysbio/syy032 (2018). 10.1093/sysbio/syy032 PMC6101584 29718447

