# Supplementary material for: Adaptive evolution and phylogeny of cerithioid gastropods with six new mitogenomes
Source: Sci Rep. 2025 Dec 2;16:718. doi: 10.1038/s41598-025-30310-z (PMC12780104; doi:10.1038/s41598-025-30310-z)

Table S1. List of species of Cerithioidea used in phylogenetic analyses based on mitogenomes

Family	Species	Locality	GenBank Acc.	Reference
Batillariidae	*Batillaria attramentaria* (US)		NC_047187	Group et al. (2019)
	Batillaria attramentaria* (KR)	Yeosu, Jeonnam, Korea	PV619093	This study
	Batillaria cumingii		MT323103	Yan et al. (2020a)
	Batillaria multiformis*	Kangjin, Jeonnam, Korea	PV619094	This study
	Batillaria zonalis		MT363252	Yan et al. (2020b)
Cerithiidae	*Clypeomorus* sp.		PQ310514	Unpublished
Hemisinidae	*Aylacostoma chloroticum*		PQ279514	Forestello et al. (2025)
Turritellidae	*Maoricolpus roseus*		NC_068097	Unpublished
	Turritella bacillum		NC_029717	Zeng et al. (2016)
Pachychilidae	*Tylomelania sarasinorum*		NC_030263	Hilgers et al. (2016)
Paludomidae	*Pseudocleopatra dartevellei*		NC_045095	Stelbrink et al. (2019)
Pleuroceridae	*Leptoxis ampla*		KT153076	Whelan and Strong (2016)
Potamididae	***Cerithideopsis largillierti****	Ongjin, Incheon, Korea	PV619097	This study
	Cerithidea sinensis		KY021067	Xu et al. (2019)
	Cerithidea obtusa		NC_039951	Nguyen et al. (2018)
	Cerithidea rhizophorarum*	Boseong, Jeonnam, Korea	PV619096	This study
	Cerithidea tonkiniana (CH)		MZ168697	Yang and Deng (2022)
	Cerithidea tonkiniana* (KR)	Goheung, Jeonnam, Korea	PV619095	This study
	Pirenella pupiformis		LC648322	Kato et al. (2022)
	Pirenella cingulata*	Hadong, Gyeongnam, Korea	PV619098	This study
Semisulcospiridae	*Semisulcospira coreana*		NC_037771	Kim and Lee (2018)
	Semisulcospira egretta		PQ165124	Xu et al. (2025)
	Semisulcospira gottschei		MK559478	Lee et al. (2019)
	Semisulcospira libertina		NC_023364	Zeng et al. (2015)
	Semisulcospira ningpoensis		OR911921	Unpublished
	Koreoleptoxis nodifila		NC_046494	Choi et al. (2021)
	Koreoleptoxis amurensis		PQ334808	Unpublished
	Koreoleptoxis friniana		NC_084162	Unpublished
	Koreoleptoxis globus ovalis		LC006055	Unpublished
	Hua aristarchorum		NC_085155	Xu et al. (2024)
Thiaridae	*Melanoides tuberculata*		MZ321058	Ling et al. (2022)
	Tarebia granifera		MZ662113	Yin et al. (2022)
	Mieniplotia scabra		PQ327780	Unpublished
Ampullariidae (Ampullarioidea)	*Pila virens*		NC_077599	Unpublished
	Pomacea canaliculata		NC_024586	Zhou et al. (2016)

An asterisk(*) occurs in the mitochondrial genome sequences of the species appended in this study.

Table S2. Annotation table for the mitochondrial genome of *Batillaria attramentaria*

Gene	Direction	Position		Size	Intergenic Nucleotides	Codon		Anti-codon
		From	To			Start	Stop	
cox1	+	1	1533	1533	53	ATG	TAA	
cox2	+	1587	2276	690	37	ATG	TAA	
trnS2	+	2314	2380	67	86			TGA
nad4l	+	2467	2757	291	-7	ATG	TAA	
nad4	+	2751	4118	1368	2	ATG	TAA	
trnH	+	4121	4185	65	0			GTG
nad5	+	4186	5904	1719	135	ATG	TAA	
trnF	+	6040	6106	67	318			GAA
trnC	-	6425	6489	65	33			GCA
trnA	-	6523	6589	67	72			TGC
trnN	-	6662	6728	67	28			GTT
trnW	-	6757	6823	67	7			TCA
trnE	-	6831	6902	72	22			TTC
trnY	-	6925	6991	67	26			GTA
trnR	-	7018	7083	66	4			TCG
trnQ	-	7088	7153	66	3			TTG
trnK	-	7157	7223	67	41			TTT
cox3	-	7265	8044	780	3	ATG	TAA	
trnM	-	8048	8114	67	14			CAT
cytb	-	8129	9268	1140	-47	ATG	TAG	
nad6	-	9222	9773	552	6	ATG	TAA	
trnP	-	9780	9846	67	0			TGG
nad1	-	9847	10785	939	0	ATG	TAG	
trnL2	-	10786	10852	67	21			TAA
trnL1	-	10874	10943	70	0			TAG
rrnL	-	10944	12315	1372	4			
trnV	-	12320	12386	67	9			TAC
trnG	-	12396	12462	67	13			TCC
trnT	-	12467	12536	70	0			TGT
rrnS	-	12537	13437	901	71			
trnS1	+	13509	13575	67	0			GCT
nad2	+	13576	14643	1068	3	ATG	TAG	
trnD	+	14647	14715	69	1			GTC
atp8	+	14717	14893	177	55	ATG	TAA	
atp6	+	14949	15644	696	20	ATG	TAA	
trnI	+	15665	15735	71	0			GAT
nad3	+	15736	16089	354	9	ATG	TAA	

Table S3. Annotation table for the mitochondrial genome of *Batillaria multiformis*

Gene	Direction	Position		Size	Intergenic Nucleotides	Codon		Anti-codon
		From	To			Start	Stop	
cox1	+	1	1533	1533	59	ATG	TAA	
cox2	+	1593	2282	690	37	ATG	TAG	
trnS2	+	2320	2386	67	99			TGA
nad4l	+	2486	2776	291	-7	ATG	TAG	
nad4	+	2770	4137	1368	2	ATG	TAA	
trnH	+	4140	4204	65	0			GTG
nad5	+	4205	5923	1719	148	ATG	TAA	
trnF	+	6072	6138	67	400			GAA
trnC	-	6539	6603	65	32			GCA
trnA	-	6636	6702	67	73			TGC
trnN	-	6776	6842	67	25			GTT
trnW	-	6868	6934	67	10			TCA
trnE	-	6945	7016	72	22			TTC
trnY	-	7039	7105	67	26			GTA
trnR	-	7132	7197	66	4			TCG
trnQ	-	7202	7267	66	3			TTG
trnK	-	7271	7337	67	43			TTT
cox3	-	7381	8160	780	3	ATG	TAA	
trnM	-	8164	8230	67	14			CAT
cytb	-	8245	9384	1140	-47	ATG	TAG	
nad6	-	9338	9889	552	6	ATG	TAA	
trnP	-	9896	9962	67	0			TGG
nad1	-	9963	10901	939	0	ATG	TAA	
trnL2	-	10902	10968	67	21			TAA
trnL1	-	10990	11059	70	0			TAG
rrnL	-	11060	12431	1372	5			
trnV	-	12436	12502	67	9			TAC
trnG	-	12512	12578	67	4			TCC
trnT	-	12583	12652	70	0			TGT
rrnS	-	12653	13555	973	71			
trnS1	+	13626	13692	67	0			GCT
nad2	+	13693	14760	1068	3	ATG	TAG	
trnD	+	14764	14832	69	1			GTC
atp8	+	14834	15010	177	58	ATG	TAA	
atp6	+	15069	15764	696	20	ATG	TAA	
trnI	+	15785	15855	71	0			GAT
nad3	+	15856	16209	354	9	ATG	TAA	

Table S4. Annotation for the mitochondrial genome of *Cerithioidea tonkiniana*

**Gene**	**Direction**	**Position**		**Size**	**Intergenic Nucleotides**	**Codon**		**Anti-codon**
		From	To			Start	Stop	
cox1	+	1	1533	1533	42	ATG	TAG	
cox2	+	1576	2265	690	28	ATG	TAA	
trnS2	+	2294	2359	66	2			TGA
trnQ	+	2362	2430	69	12			TTG
nad4l	+	2443	2733	291	-7	ATG	TAG	
nad4	+	2727	4094	1368	9	ATG	TAA	
trnH	+	4104	4168	65	0			GTG
nad5	+	4169	5887	1719	0	ATG	TAG	
trnF	+	5888	5958	71	313			GAA
trnC	-	6272	6333	62	47			GCA
trnR	-	6381	6449	69	6			TCG
trnA	-	6456	6524	69	9			TGC
trnN	-	6534	6601	68	8			GTT
trnW	-	6610	6676	67	4			TCA
trnE	-	6681	6749	69	3			TTC
trnY	-	6753	6819	67	17			GTA
trnK	-	6837	6902	66	29			TTT
cox3	-	6932	7711	780	3	ATG	TAA	
trnM	-	7715	7782	68	13			CAT
cytb	-	7796	8935	1140	-38	ATG	TAA	
nad6	-	8898	9440	543	2	ATG	TAA	
trnP	-	9443	9508	66	1			TGG
nad1	-	9510	10448	939	0	ATG	TAA	
trnL2	-	10449	10515	67	3			TAA
trnL1	-	10519	10589	71	0			TAG
rrnL	-	10590	11950	1361	0			
trnV	-	11951	12019	69	7			TAC
trnG	-	12027	12094	68	6			TCC
trnT	-	12101	12171	71	0			TGT
rrnS	-	12172	13047	876	65			
trnS1	+	13112	13178	67	0			GCT
nad2	+	13179	14246	1068	5	ATG	TAA	
trnD	+	14252	14320	69	3			GTC
atp8	+	14324	14485	162	20	ATG	TAA	
atp6	+	14506	15201	696	6	ATG	TAA	
trnI	+	15208	15276	69	1			GAT
nad3	+	15278	15631	354	8	ATG	TAA	

Table S5. Annotation table for the mitochondrial genome of C*erithioidea rhizophorarum.*

Gene	Direction	Position		Size	Intergenic Nucleotides	Codon		Anti-codon
		From	To			Start	Stop	
cox1	+	1	1533	1533	46	ATG	TAA	
cox2	+	1580	2269	690	31	ATG	TAA	
trnS2	+	2301	2368	68	3			TGA
trnQ	+	2372	2439	68	11			TTG
nad4l	+	2451	2741	291	-7	ATG	TAG	
nad4	+	2735	4102	1368	9	ATG	TAG	
trnH	+	4112	4176	65	0			GTG
nad5	+	4177	5895	1719	0	ATG	TAA	
trnF	+	5896	5966	71	322			GAA
trnC	-	6289	6350	62	38			GCA
trnR	-	6389	6455	67	6			TCG
trnA	-	6462	6530	69	25			TGC
trnN	-	6556	6623	68	7			GTT
trnW	-	6631	6697	67	4			TCA
trnE	-	6702	6771	70	3			TTC
trnY	-	6775	6840	66	18			GTA
trnK	-	6859	6926	68	41			TTT
cox3	-	6968	7747	780	3	ATG	TAA	
trnM	-	7751	7818	68	12			CAT
cytb	-	7831	8970	1140	-38	ATG	TAA	
nad6	-	8933	9475	543	2	ATT	TAA	
trnP	-	9478	9543	66	0			TGG
nad1	-	9545	10483	939	0	ATG	TAA	
trnL2	-	10484	10550	67	6			TAA
trnL1	-	10557	10627	71	0			TAG
rrnL	-	10628	11990	1363	0			
trnV	-	11991	12059	69	3			TAC
trnG	-	12063	12128	66	12			TCC
trnT	-	12141	12211	71	0			TGT
rrnS	-	12212	13095	886	66			
trnS1	+	13161	13227	67	0			GCT
nad2	+	13228	14295	1068	3	ATG	TAA	
trnD	+	14299	14367	69	3			GTC
atp8	+	14371	14532	162	0	ATG	TAA	
atp6	+	14553	15248	696	6	ATG	TAA	
trnI	+	15255	15323	69	1			GAT
nad3	+	15325	15678	354	9	ATG	TAA	

Table S6. Annotation table for the mitochondrial genome of *Cerithideopsis largillierti.*

Gene	Direction	Position		Size	Intergenic Nucleotides	Codon		Anti-codon
		From	To			Start	Stop	
cox1	+	1	1533	1533	33	ATG	TAA	
cox2	+	1567	2256	690	22	ATG	TAA	
trnS2	+	2279	2346	68	1			TGA
trnQ	+	2348	2415	68	9			TTG
nad4l	+	2425	2715	291	-7	ATG	TAG	
nad4	+	2709	4076	1368	0	ATG	TAA	
trnH	+	4077	4142	66	0			GTG
nad5	+	4143	5861	1719	4	ATG	TAA	
trnF	+	5866	5934	69	249			GAA
trnC	-	6184	6248	65	29			GCA
trnR	-	6278	6344	67	17			TCG
trnA	-	6362	6429	68	9			TGC
trnN	-	6439	6505	67	6			GTT
trnW	-	6512	6580	69	4			TCA
trnE	-	6585	6650	66	1			TTC
trnY	-	6652	6718	67	6			GTA
trnK	-	6725	6791	67	36			TTT
cox3	-	6828	7607	780	3	ATG	TAA	
trnM	-	7611	7677	67	7			CAT
cytb	-	7685	8824	1140	-38	ATG	TAA	
nad6	-	8787	9329	543	2	ATG	TAA	
trnP	-	9332	9396	65	0			TGG
nad1	-	9397	10335	939	0	ATG	TAA	
trnL2	-	10336	10402	67	0			TAA
trnL1	-	10403	10473	71	0			TAG
rrnL	-	10474	11832	1359	0			
trnV	-	11833	11901	69	3			TAC
trnG	-	11905	11971	67	4			TCC
trnT	-	11976	12044	69	0			TGT
rrnS	-	12045	12921	877	67			
trnS1	+	12988	13054	67	0			GCT
nad2	+	13055	14122	1068	4	ATG	TAA	
trnD	+	14127	14194	68	3			GTC
atp8	+	14198	14359	162	37	ATG	TAA	
atp6	+	14397	15092	696	9	ATG	TAA	
trnI	+	15102	15170	69	1			GAT
nad3	+	15172	15525	354	6	ATG	TAA	

Table S7. Annotation table for the mitochondrial genome of *Pirenella cingulata*

Gene	Direction	Position		Size	Intergenic Nucleotides	Codon		Anti-codon
		From	To			Start	Stop	
cox1	+	1	1533	1533	34	ATG	TAA	
cox2	+	1568	2257	690	24	ATG	TAA	
trnS2	+	2282	2348	67	0			TGA
trnQ	+	2349	2416	68	11			TTG
nad4l	+	2428	2718	291	-7	ATA	TAG	
nad4	+	2712	4079	1368	3	GTG	TAA	
trnH	+	4083	4148	66	0			GTG
nad5	+	4149	5867	1719	31	ATG	TAA	
trnF	+	5899	5968	69	263			GAA
trnC	-	6231	6294	64	38			GCA
trnR	-	6333	6398	66	19			TCG
trnA	-	6418	6485	68	16			TGC
trnN	-	6502	6569	68	2			GTT
trnW	-	6572	6640	69	9			TCA
trnE	-	6650	6716	67	2			TTC
trnY	-	6719	6784	66	8			GTA
trnK	-	6793	6861	69	39			TTT
cox3	-	6901	7680	780	3	ATG	TAA	
trnM	-	7684	7750	67	22			CAT
cytb	-	7773	8912	1140	-1	ATG	TAG	
nad6	-	8912	9418	507	2	ATG	TAA	
trnP	-	9421	9486	66	0			TGG
nad1	-	9487	10425	939	0	ATG	TAG	
trnL2	-	10426	10493	68	5			TAA
trnL1	-	10499	10569	71	0			TAG
rrnL	-	10570	11927	1358	0			
trnV	-	11928	11995	68	15			TAC
trnG	-	12011	12077	67	3			TCC
trnT	-	12081	12150	70	0			TGT
rrnS	-	12151	13035	885	65			
trnS1	+	13100	13166	67	0			GCT
nad2	+	13167	14234	1068	4	ATG	TAA	
trnD	+	14239	14309	71	0			GTC
atp8	+	14310	14471	162	27	ATG	TAA	
atp6	+	14499	15194	696	6	ATG	TAA	
trnI	+	15201	15269	69	1			GAT
nad3	+	15271	15624	354	5	ATG	TAA	

Fig. S1 tRNA structure of *Batillaria attramentaria*

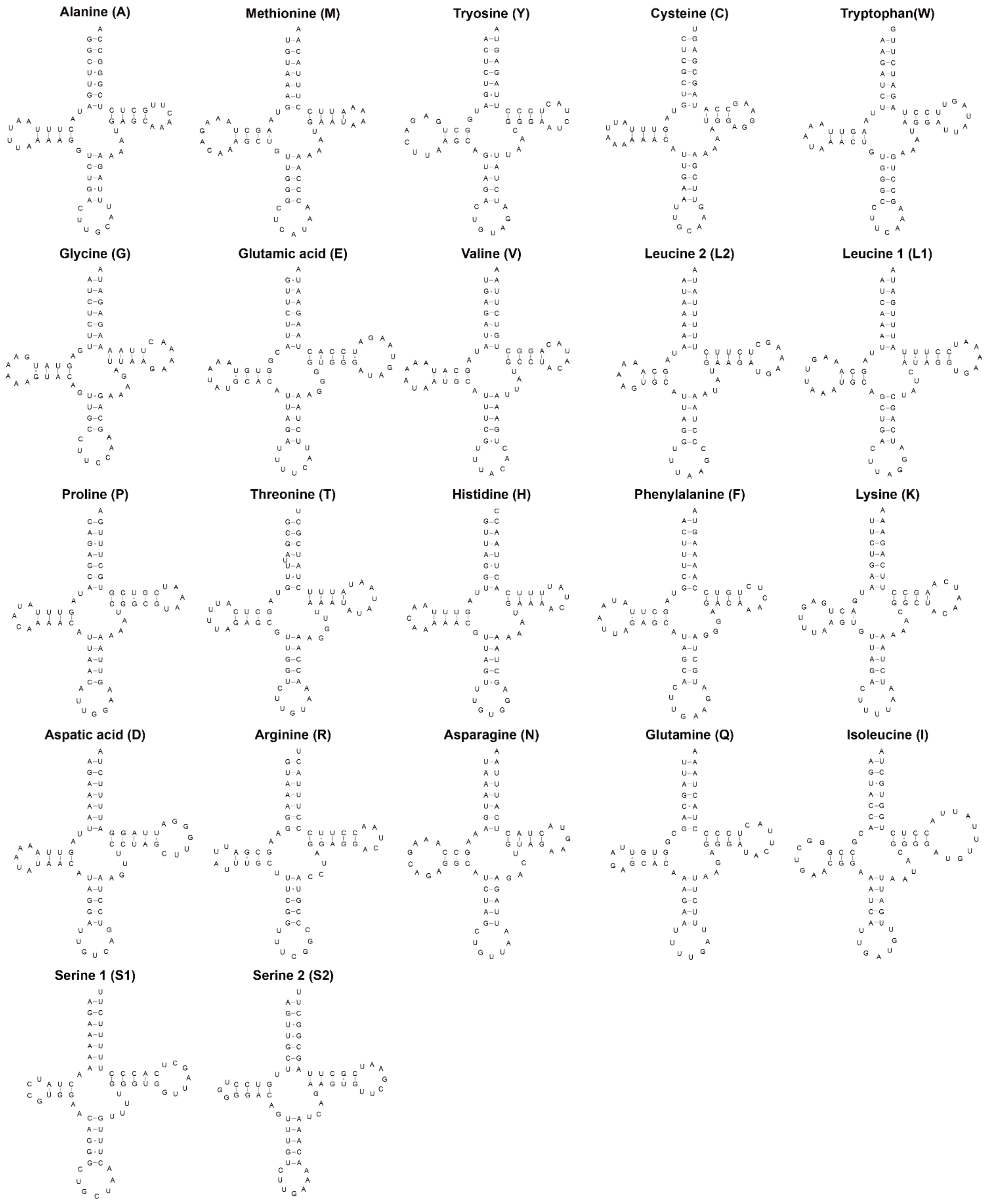

Fig. S2 tRNA structure of *Batillaria multiformis*

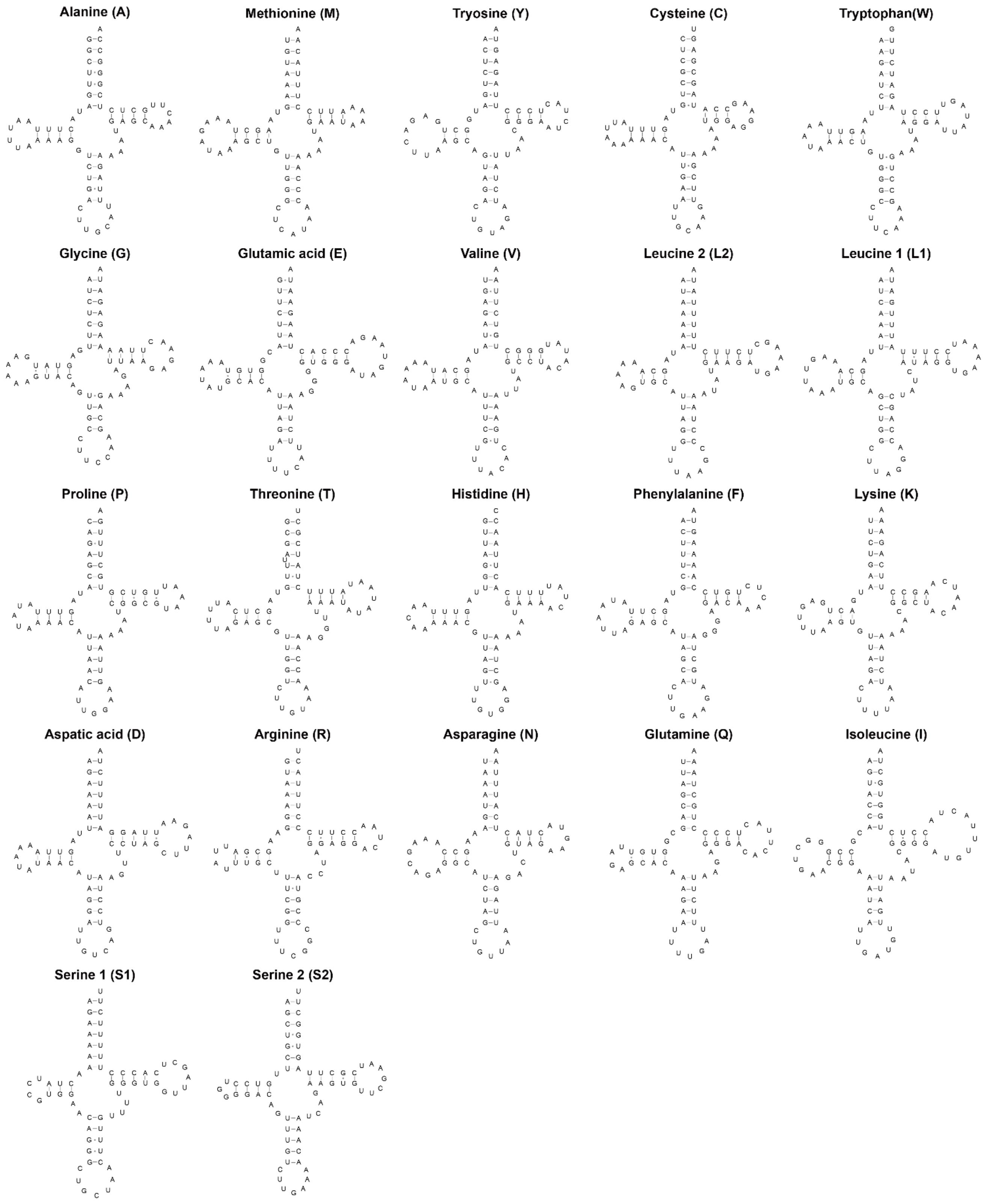

Fig. S3 tRNA structure of *Cerithidea tonkiniana*

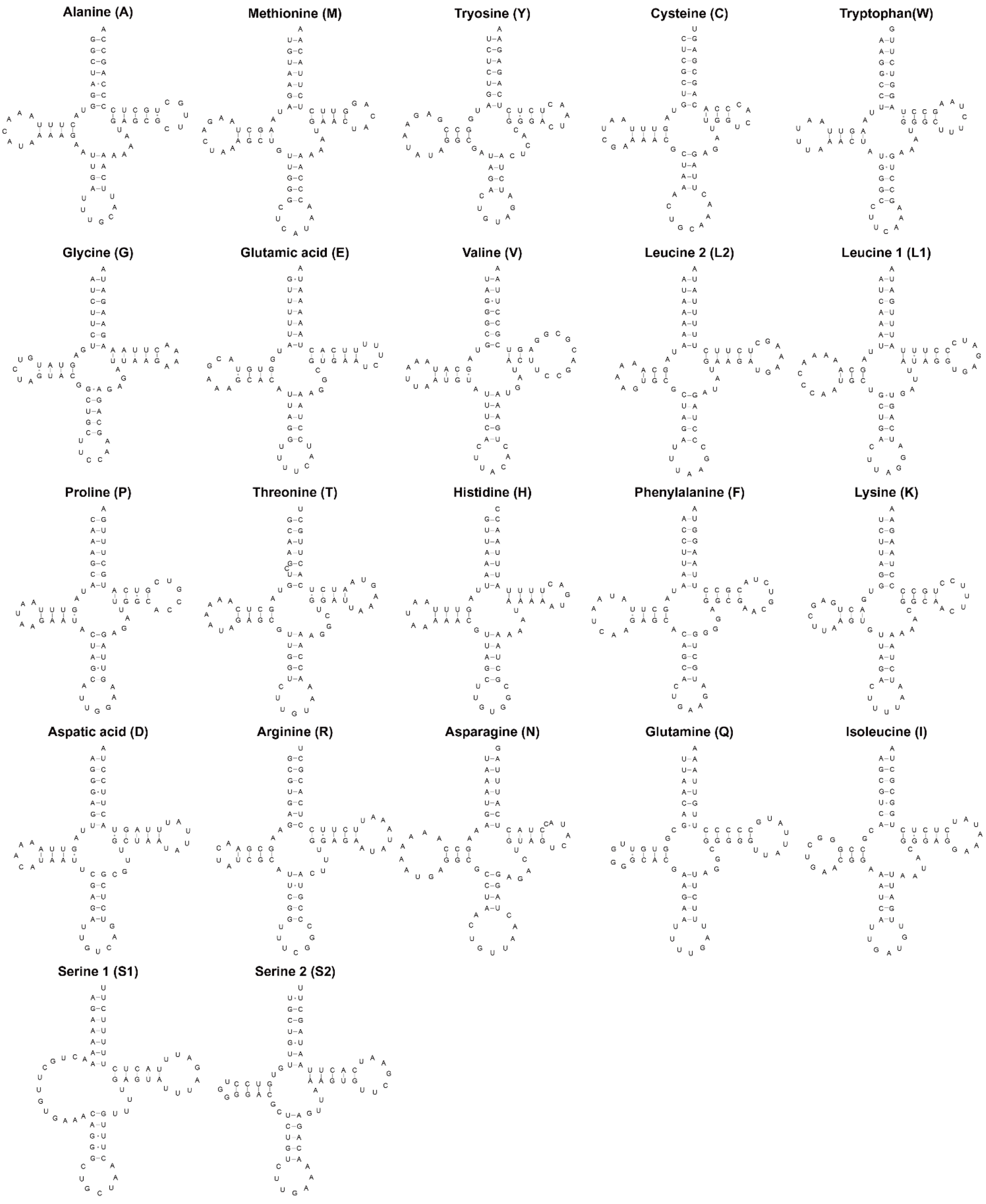

Fig. S4 tRNA structure of *Cerithidea rhizophorarum*

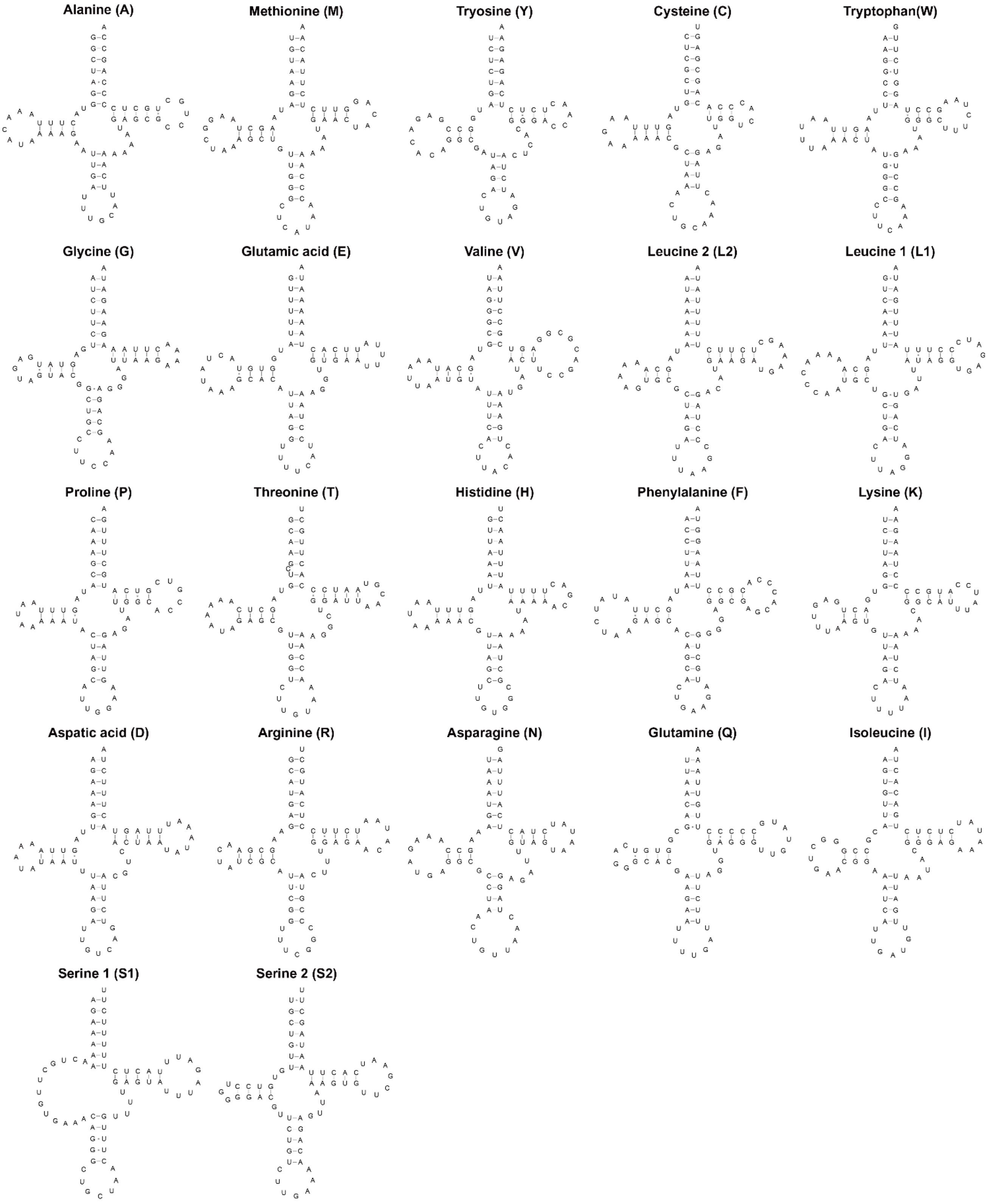

Fig. S5 tRNA structure of *Cerithideopsis largillierti*

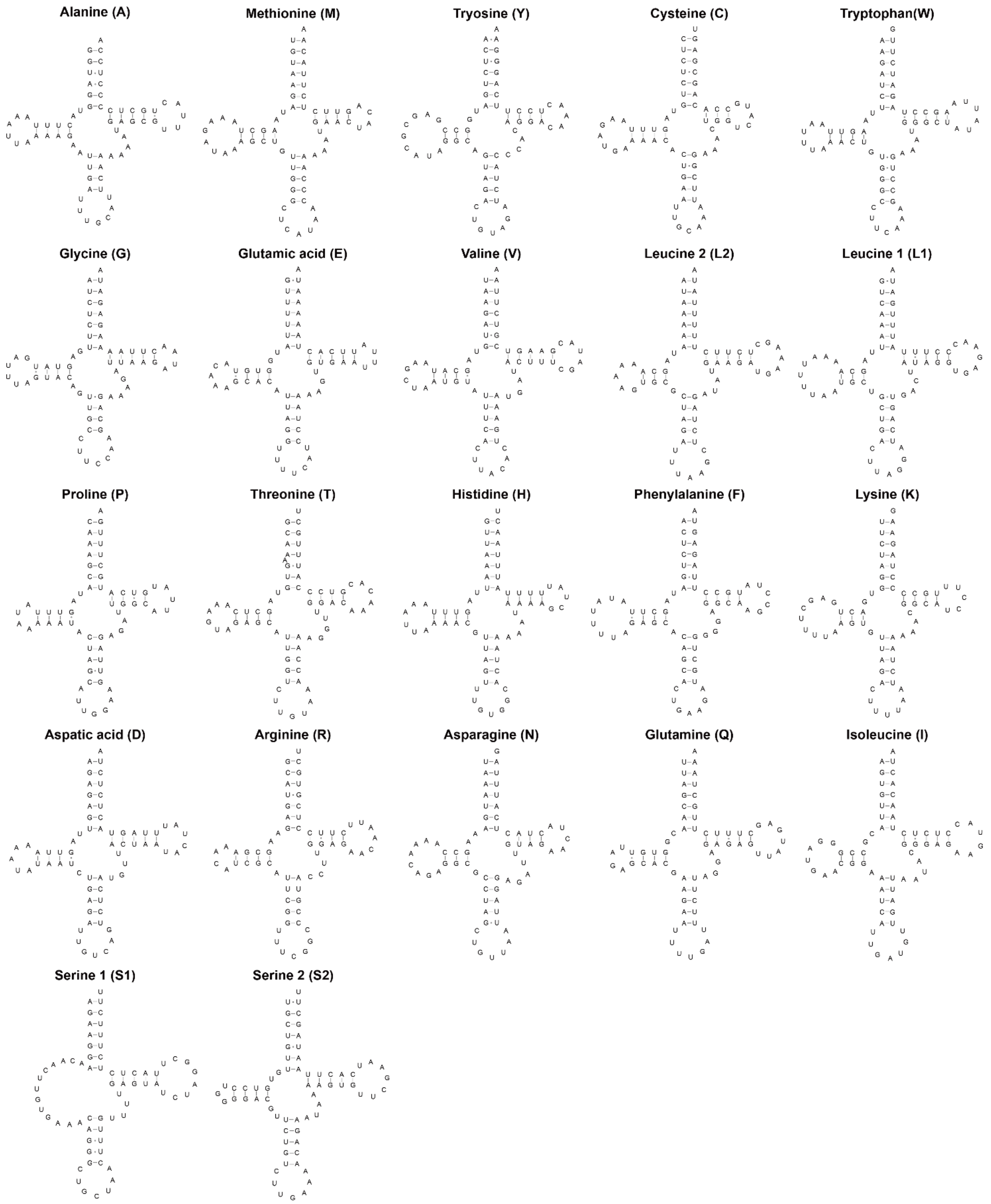

Fig. S6 tRNA structure of *Pirenella cingulata*

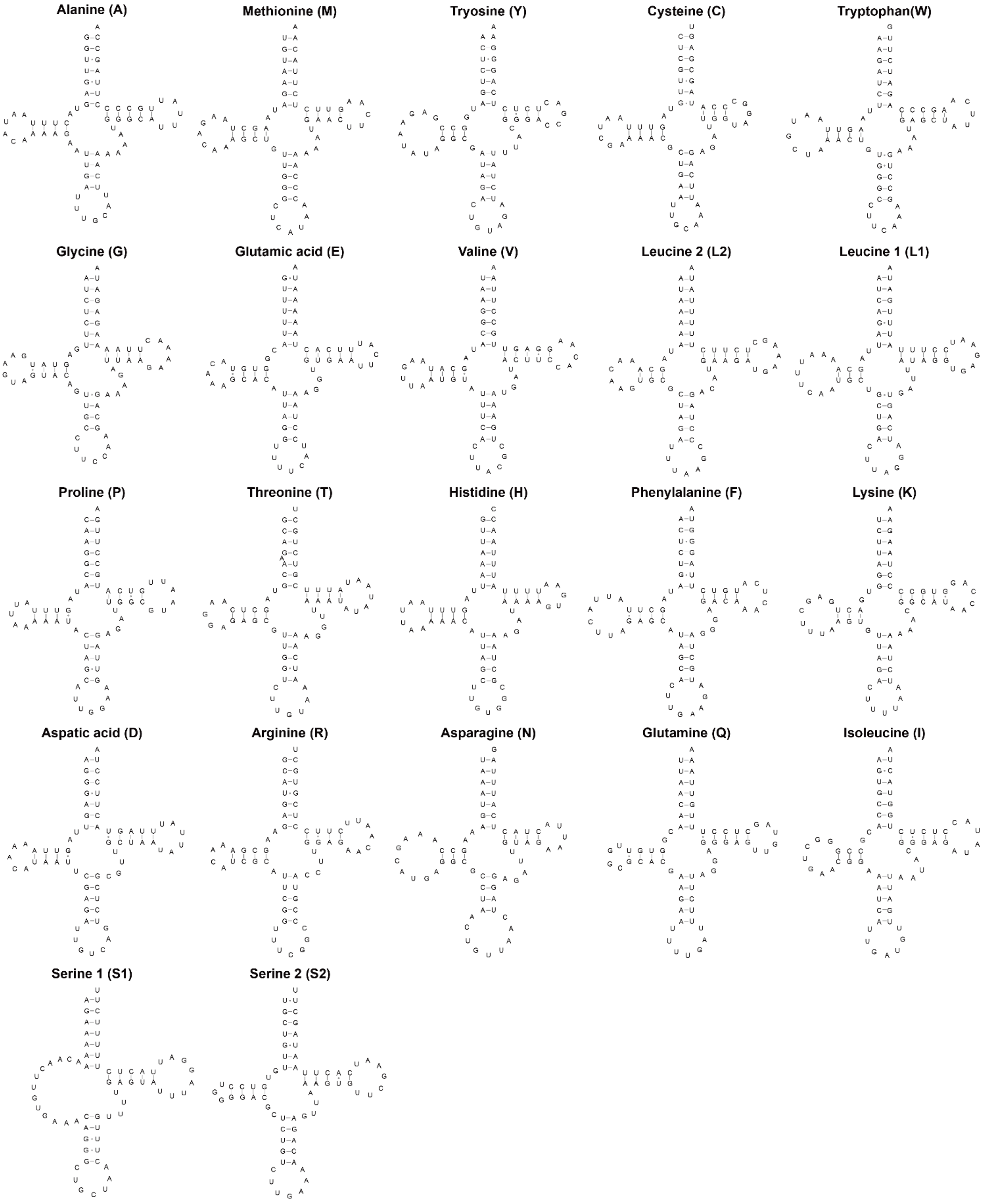

Fig. S7 ML and BI tree of Cerithioidea based on 13PCGs nucleotide and amino acids datasets

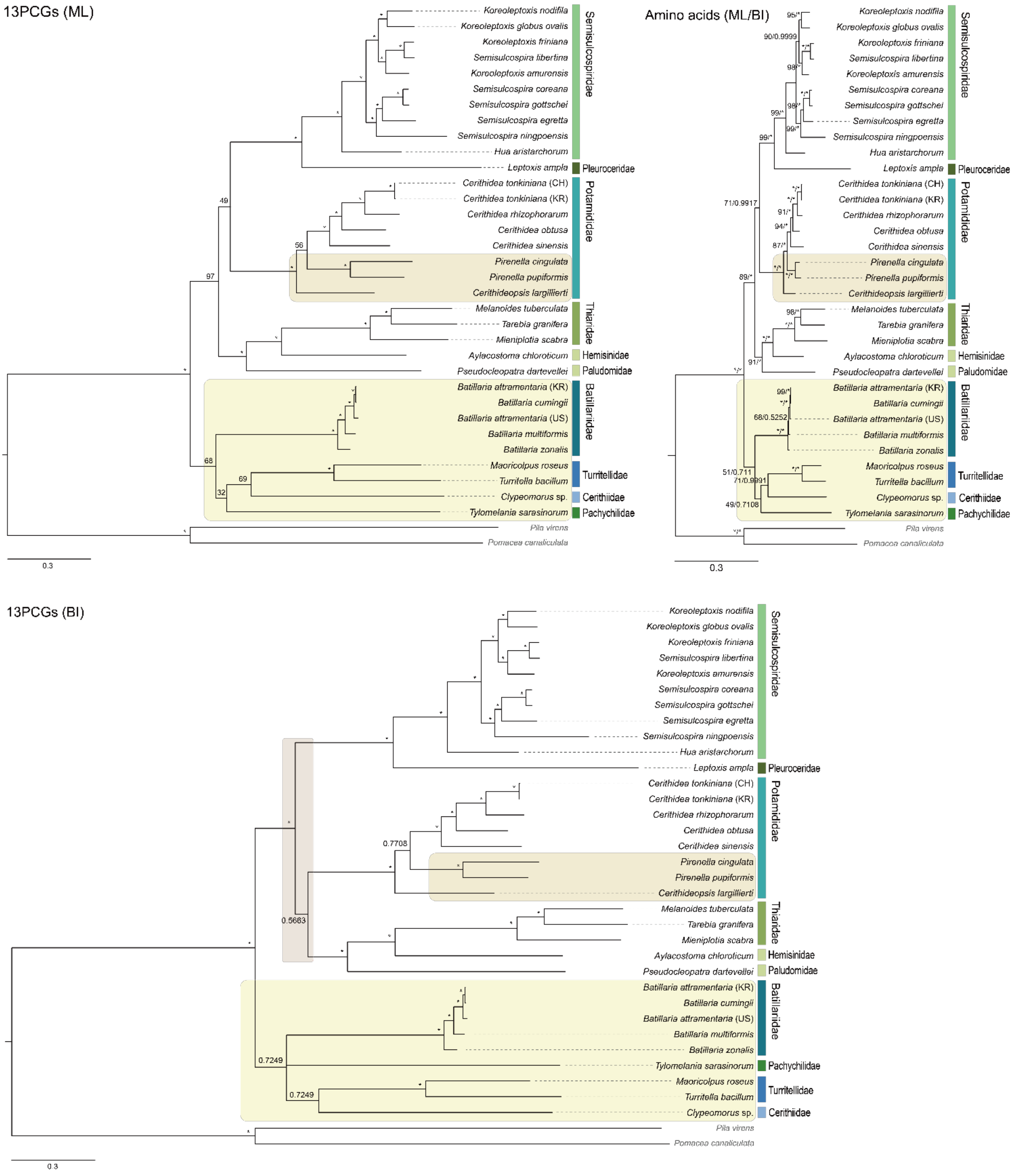

Supplement: Supplementary file 1 — Supplementary Information. [file 41598_2025_30310_MOESM1_ESM.pdf]
